# Discourse on the Life, Character, and Services of Dr. Daniel Drake

**Published:** 1853-02

**Authors:** S. D. Gross

**Affiliations:** Professor of Surgery


### WESTERN JOURNAL

OF

### MEDICINE AND SURGERY.

FEBRUARY, 1853.

Art. I.—*A Discourse on the Life, Character, and Services of Daniel Drake, M. D.*—Delivered by request, before the Faculty and Medical Students of the University of Louisville, January 27, 1853. By S. D. Gross, M. D., Professor of Surgery.

On the 5th of November, 1852, a telegraphic dispatch, dated at Cincinnati at 3 o'clock in the afternoon, and received in this city three hours afterwards, announced to us that our profession and our country were about to be deprived of a great and good man. It was short but unmistakable, full of significance and foreboding. "Dr. Drake is supposed to be dying." Another message, received the next morning at 10 o'clock, only served to confirm the sad and melancholy intelligence of the preceding evening. News, so entirely unexpected, fell with stunning effect upon the heart and intellect of the friends of the departed physician in this community, so long the theatre of his active and useful labors. It is true, it had been rumored that he was unwell, somewhat indisposed, but no one had thought him seriously ill, much less despaired of his life. Hence, when the intelligence of his demise reached us, it took every one by surprise. His intimate friends and acquaintances, those who knew and loved him best, had never permitted themselves to think of him in such a connexion; they had hoped and prayed that he might have length of days, and that he might be spared, long enough at least, to complete the great work which had so long and so intensely occupied his mind, and which now only waited. as it were, to receive the last finishing touches of his pen. They had hoped that God would vouchsafe him health and life that he might achieve the great and now only remaining object of his ambition, and that he might thus be permitted to hand to his professional brethren, for their benefit, and the benefit of his race, the record of his acts, and the bond of his devotion to the great and noble pursuit which had so long occupied his thoughts and affections, and engaged the best energies of his mind and body.

Only a fortnight before they had seen him in their very midst, the "observed of all observers," almost the "gayest of the gay." At the meeting in this city, on the 21st of October, of the Kentucky State Medical Society, whose honored guest he was, he looked so well that every one was struck with the circumstance; and at the anniversary supper, two evenings afterwards, he responded, in terms of glowing eloquence, to a complimentary toast. On the following morning, with steps that were never more light, and spirits that were never more buoyant, he called upon a number of his friends, as well as upon his former colleagues in this University, prior to the departure of the Cincinnati packet, which was to convey him, as it proved, for the last time, upon the bosom of the Ohio. Little did we think, as we shook hands, that we had met together for the last time, and that the separation which was about to take place was to be forever. How little does man know the future, how incompetent is he to lift the veil which screens him from his destiny! It was only a few hours before his departure that he paid his respects to one of his former colleagues, who still lingers among us, bowed down by the frosts and labors of more than eighty winters. While sitting with him, and rapidly talking over the topics of the day, he was painfully impressed with the changes which time and disease had wrought upon him since their last interview, and on returning, soon after, to his lodgings, he could not refrain from mentioning the circumstance to a female friend, and expressing his conviction that he should never again behold him. Strange prophecy! The one still lives, clinging like an ancient and venerable ivy to the tree of time, while the other, many years his junior, lies cold and silent in the winding-sheet of death.

The wanderer is not long in performing his journey. A few hours are sufficient to restore him to his home and to the bosom of his children and grand-children, who, as they see his familiar face, cluster around him, welcoming him with their smiles and their affection. He has finished his last journey on earth; he has gazed for the last time upon the beautiful scenery of his beloved Ohio, enhanced a thousand fold by the Great Portrait Painter and Chemist of Nature. Never did the foliage of the forest, adorned and diversified by the endless and ever-changing tints of autumn, present itself in so attractive and resplendent a form. As he looked upon it, his mind involuntarily recurred to the period of his childhood, when, surrounded by his parents, his brothers and sisters, he dwelt in the wilds of Kentucky, with nothing but trees, and birds, and squirrels, and wild-flowers, for his playmates and companions. The scene revived in him the recollection of his early struggles, his hopes, and aspirations, and, perhaps, admonished him, as he silently connected the present with the past, that the "sear and yellow leaf of autumn" is a fit emblem of man's mortality, and of the evanescent, transitory character of his earthly existence.

The tolling of the bell, that solemn messenger of God, awakens the great physician from the revery into which he has lapsed, now that the first greetings of the happy family group are over, and with a light heart he goes forth to offer up, for the last time, his orisons at the altar of his Savior. His last Sabbath, but one, upon the earth is passed, and the week, thus auspiciously begun, is spent, at least in part, in the midst of his pupils, at the bed-side of the sick poor at the Hospital, and in his private study, distilling from the alembic of his brain thoughts and words of mighty import to his professional brethren. His fellow citizens are assembled to do honor to the memory of America's "Great Secretary;" and, inspired by the occasion, at once solemn and impressive, he makes his last public speech, calling the attention of the young men around him to Daniel Webster's dying declaration of the inestimable value of the christian religion, and of man's utter dependance upon Divine Mercy. "Who," he remarked "shall say that the simple utterance of the departed statesman—'Thy rod, Thy rod—Thy staff, Thy staff, they comfort me'—do not constitute the greatest act of that life of great acts?"

His couch that night knows no repose; the hand of disease is laid heavily upon his brow, and to-morrow's sun finds him weary and unrefreshed. Thus a few days are passed, the enemy now receding, and now advancing, until, at length, it is but too evident to both patient and friends that the hour of convalescence, if it is ever to come, is far off. Gradually but steadily the destroyer progresses in his work, making sure and fearful inroads upon the system; great debility ensues; the brain is no longer capable of shedding its wonted light; thought flows sluggishly and reluctantly; speech has lost its facility of utterance; and the sufferer is oppressed by a sense of annihilation, indescribable and overwhelming, and attended with the most terrible despondency. He still sees and talks, but is hardly able to think or feel! Rousing himself from his lethargy, he beckons to one near and dear to him, and speaks to him concerning the unfinished condition of his great work, saying that his only ambition was to complete it, and expressing a hope that God might spare him for that end. Again he relapses into a state of torpor; his agony is so intense that he prays to be released; he has no longer any desire to live; all schemes of ambition, even the wish to finish his work, have passed from his mind; his soul is chastened and purified; God has taught him the folly of all earthly hopes, and the vanity of all human expectations. While the mind and body are thus oppressed and palsied, unstrung and tortured, the soul is buoyed up with hope and joy, and clings with pertinacious tenacity to its Savior. "Every nerve is strung with the most intense desire to hold Him fast."

All of a sudden the sufferer expresses himself better; he experiences "temporary relief;" an anodyne draught is administered, and presently he falls into a sleep so sweet and natural that the watchers think of approaching convalescence. Vain and delusive hope! The sleep so "sweet and natural" is the sleep of death; life is flickering in its socket, and just before it is extinguished the eyes once more open and beam with an unearthly radiance, as the sun sometimes after a cloudy day suddenly bursts through the mist, and illumines for an instant the horizon before he finally sinks into the dark shades of night. The spirit had fled so gently and so softly that the precise moment of its departure was hardly perceptible. The silver cord was loosed, the golden bowl was broken, the duty of the watchers and the physicians was over, and the mourners went about the streets.

The news of the melancholy event spread with the lightning's speed. Every where in the profession, as well as in the nation at large, it excited the most lively emotions of sorrow. The Medical Schools of Cincinnati and the University of Louisville suspended their lectures, and passed resolutions, expressive of their regret and of their great loss, as well as of their sympathy with the family of the deceased. Numerous medical societies in the South-west also held meetings, and gave vent to their feelings in appropriate resolutions. The whole profession, in fact, felt that it had lost one of its best and greatest men.

The disease which deprived him of his life, and which struck from the firmament of our profession one of its brightest ornaments, was congestion of the brain, or, perhaps, as I am inclined to think from the character of the symptoms, arachnitis. He had long dreaded the effects of this malady, the first seizure of which he experienced while a professor at Lexington in 1825, and which came very nigh proving fatal to him. Nearly six weeks elapsed before his health was sufficiently re-established to enable him to resume his duties in the University. He was ever afterwards subject to occasional attacks of the disease, but they were generally mild, and usually passed off in a few days under the influence of simple remedies.

The immediate cause of his last illness was over exertion of the brain, produced by the labors and excitement consequent upon the opening of the session of the Medical College of Ohio. His toils, always excessive, were unceasing; his vigilance never slumbered; aware that much of the prosperity of the school would necessarily depend upon his personal exertion, he spared no effort to insure its success, to advance its dignity, and to promote its interests. He knew that the eyes of the profession of the whole South-west were upon him, and that his character as the re-builder of the Medical College of Ohio, of which he had been the founder more than thirty years ago, was at stake. His nervous system was strung to the utmost, and it was while thus occupied that he was seized with the malady which was destined to destroy him.

His illness was of short duration; death called him hence before disease had worn out his frame, or age dimmed his intellectual powers. The week before his final seizure he lectured and wrote with his accustomed energy and ability. Like a green leaf in autumn, loosened by the frost, and shaken by the wind, he fell prematurely from the tree of life, which he might, according to man's feeble judgment, have yet adorned for many years. At the time of his death he had just completed his sixty-seventh year.

His funeral, delayed by the unexpected and unavoidable detention of his only son, Charles D. Drake, Esq., of St. Louis, took place on Wednesday afternoon, November 10th, and was attended by his late colleagues and pupils, by the Faculties and pupils of the other Cincinnati schools of medicine, and by an immense concourse of citizens. The procession was long and solemn, and the grief which was depicted upon every countenance afforded a striking demonstration of the esteem and affection of the people among whom he had lived upwards of half a century, and who knew so well how to appreciate his many virtues. The profession felt that they had lost their brightest ornament, and the citizens of Cincinnati that they had been deprived of one of their conscript fathers. The body was placed in a vault in the Episcopal burying-ground. Next day, attended only by his family and a few very intimate friends, it was removed to Spring Grove Cemetery, and deposited, in fulfilment of his own wishes, by the side of the remains of her, who, for eighteen years, had been the worthy sharer of his joys and his sorrows, and whose memory he romantically cherished during a period of upwards of a quarter of a century.

Dr. Drake was born at Plainfield, a small village in Essex county, New Jersey, October 20th, 1785. Here he spent the first two years and a half of his life. At the expiration of this time, his father emigrated to Kentucky, then only nine years older than his son, and settled at Mays Lick, twelve miles south-west of Maysville, and fifty three miles from Lexington. Mays Lick was a colony of East-Jersey people, with a few stragglers from Virginia and Maryland; and consisted, at the period referred to, of fifty-two persons, all poor, and illiterate. Their occupation was clearing the forest and cultivating the soil. The log cabin of that day, the residence of the Drake family, constituted an interesting feature of the landscape. As the name implies, it was built of logs, generally unhewn, with a puncheon floor below, and a clap-board floor above, a small square window without glass, a chimney of "cats and clay," and a coarse roof. It consisted generally of one apartment, which served as a sitting-room, dormitory and kitchen.

The ancestors of Dr. Drake were poor, illiterate, and unknown to fame; but they possessed the great merit of being industrious, honest, temperate and pious. To spring from such ancestors, is, as he justly observes, high descent in the sight of Heaven, if not in the estimation of man. Both his grand-fathers lived in the very midst of the battle-scenes of the revolution; one of them, Shotwell, was a member of the Society of Friends, and was, of course, a non-combatant, while the other, who had no such scruples, was frequently engaged in the partizan warfare of his native State. * The father of Dr. Drake died at Cincinnati in 1832, and the mother in 1831; both at an advanced age.

It was at Mays Lick, amidst the people whom I have briefly described, that young Drake spent the first fifteen years of his life, performing such labors as the exigencies of his family demanded. In the winter months, generally from November until March, he was sent to school, distant, usually about two miles from his father's cabin, while during the remainder of the year he worked upon the farm,

* Dr. Drake's Reminiscential Letters to his Children. These Letters, a copy of which was kindly put into my hands by the family of the deceased, will be particularly referred to hereafter.

attending to the cattle, tilling the soil, and clearing the forest, an occupation in which he always took great delight.

This kind of life, rude as it was, and uncongenial as it must have been to his taste was not without its advantages. It eminently fitted him for the observation of nature, so necessary to a physician. Nothing escaped his eye. Nature was spread out before him in all her diversified forms, and he loved to contemplate her in the majestic forest, in the mighty stream, now placid and now foaming with anger, in the green fields, in the flowers which adorn the valley and the hill, in the clouds, in the lightning and thunder, in the snow and the frost, in the tempest and the hurricane.

It had another effect. While it had the disadvantage of preventing him from pursuing a steady course of literary culture, and fitting him for the early practice of medicine, it excited in him habits of industry and attention to business, teaching him patience and self-reliance, and giving him an insight into many matters, to which the city-trained youth is a stranger.

Finally, the physical labor which he underwent there served to impart health and vigor to his constitution, and thereby contributed to produce that power of endurance which he possessed in a degree superior to that of almost every other man I have ever known.

But the settlement of Mays Lick was not without its charms and enjoyments. To the young and imaginative mind of Drake every little spot in the landscape was invested with peculiar beauty and interest. What to an ordinary observer was barren and unattractive was to him a source of never-failing gratification. In the spring and summer the surface of the earth was carpeted with the richest verdure, and embellished with myriads of wild-flowers, which, while they rendered the air redolent with fragrance, delighted the eye by their innumerable variety. The trees, those mighty denizens of the forest, were clothed in their most majestic garb, adding beauty and grandeur to the scene, enlivened by the music of birds, which thronged the woods, and constituted, along with the merry and frolicsome squirrel, the familiar companions of the early settler. "Their notes made symphony with the winds, as they played upon the green leaves, and awakened melody as when the rays of the sun fell upon the harp of Memnon, but more real, and better for the young heart." *

The scholastic advantages of young Drake, during his residence at Mays Lick, were, as I have already hinted, very limited. The teachers of the place were itinerants, of the most ordinary description, whose function was to teach spelling, reading, writing, and cyphering, as far as the rule of three, beyond which few of them were able to go. The fashion in those days was for the whole school to learn and say their lessons aloud; a practice commended by Dr. Drake in after-life, as a good exercise of the voice, and as a means of improving the lungs, and disciplining the mind for study in the midst of noise and confusion.

His first teacher was a man from the "Eastern Shore" of Maryland, an ample exponent of the state of society in that benighted region. The school house in which he was educated was fifteen by twenty feet in its dimensions, and one story high, with a wooden chimney, a puncheon floor, and a door with a latch and string. In the winter, light was admitted through oiled paper, by long openings between the logs. Glass was not to be obtained. The ordinary fee for tuition was fifteen shillings a quarter.

As to the classics, he knew nothing of them until after he began to study medicine; for the reason, first, that there were no teachers in his neighborhood competent to

*Reminiscential Letters.

impart instruction in them, and, secondly, that he was too poor to go from home. His father stipulated with his professional preceptor that he should be sent to school for six months to learn Latin; but by some great absurdity, as he observes, this was not done until he had studied for eighteen months that which, for the want of Latin, he could not comprehend. He never, I believe, studied the Greek language. In after life he acquired some knowledge of the French.

His father's library was not, as might be supposed, either large or diversified. It was, more properly speaking, select. It consisted of a family Bible, Rippon's Hymns, Watts' Hymns for children, the Pilgrim's Progress, an old romance of the days of Knight Errantry, primers, with a plate representing John Rogers at the stake, spelling books, an arithmetic, and an almanac for the new year. As he grew up he met with Guthrie's Grammar of Geography, Entick's Dictionary, Scott's Lessons, Æsops Fables, the Life of Franklin, and Lord Chesterfield's Letters to his son, the latter of which, especially, he greatly prized. A newspaper at that day was a rarity. The first one ever published in Kentucky was issued at Lexington in 1787, the year before the emigration of the Drake family. It was called the Kentucky Gazette, and was edited by John Bradford. Nearly ten years afterwards another, the Palladium, was established at Washington, four miles off, and of this a number fell occasionally into the boy's hands, always affording him much gratification.

Works of fiction he seldom read, even in after life; first, because he had not the time, and, secondly, because they always raised in him emotions so powerful that he was obliged, in a great degree, to avoid them.

During his sojourn under his father's roof, he was a close observer of the people around him, residents as well as emigrants, the latter of whom were in the habit of passing in great numbers through the settlement. He studied their manners and habits, observed their prejudices, noticed and compared their opinions, and thus acquired important knowledge of human nature. Books and book-learning alone do not serve to make up a man's education ; he must mingle with the world, and endeavor to derive from its intercourse those lessons of wisdom and practical tact which are to regulate his conduct and beautify his life.

Thus, it will be seen that his alma mater was the forest; his teacher, nature ; his class-mates, birds, and squirrels, and wild flowers. Until the commencement of his sixteenth year, when he left home to study medicine, he had never been beyond the confines of the settlement at Mays Lick, and it was not until his twentieth year, when he went to Philadelphia to attend lectures, that he saw a large city. The "Queen of the West," as Cincinnati has been since styled, was then a mere hamlet, with hardly a few thousand inhabitants. Kentucky, at that early day, had but one University, and although it was scarcely fifty miles distant from his doors, his father was too poor to send him thither.

It was to this spot, after the lapse of nearly half a century, that the boy, now in the evening of his full and perfect manhood, turns his longing eye, anxious once more to behold the home of his early childhood. He stands before the lone and primitive cabin of his father, in which used to dwell all that were near and dear to him ; the latch-string is off the door; the hearth no longer emits its accustomed light and heat ; weeds and briers grow around and obstruct the entrance; no familiar voices are heard to greet and welcome the stranger; all is still and silent as the grave in the God's acre close by. The birds no longer salute him with their merry music ; the squirrel, whose gambols he was wont to watch with such peculiar fondness when a boy, is no longer there ; even the tall and weather-beaten elm no longer greets him with his presence. All around is silence and desolation. Upon the "door cheeks" of the cabin he discovers the initials of his own name, which he had inscribed there with his rude pen-knife fifty years before, silent witnesses of the past, reluctant to be effaced by time. As he looked around, and surveyed the changes which half a century had wrought in the landscape before him, a feeling of awe and melancholy, unutterable and indescribable, seized his soul, and the sage of three score years, the medical philosopher, and the acknowledged head of his profession in the Great Valley of the Mississippi, was instantly transmuted into the boy of fifteen. Every feeling was unmanned, and tears, warm and burning, gushed from the fountains of his soul. The whole scene of his childhood was vividly before him; the manly form of his father, the meek and gentle features of his mother, the light and sportive figures of his brothers and sisters, stood forth in bold relief, and painfully reminded him of the vanity and instability of all earthly things. Of the whole family group, eight in number, which was wont to assemble around the bright and burning hearth, only one, beside himself, remained to visit that tenantless and desolate friend of his childhood.

Young Drake was early destined for the medical profession. His father and Dr. Goforth, who afterwards became his preceptor, emigrated together from New Jersey to the West, and formed a great intimacy on their voyage down the Ohio. Although his father thought him on many points a very weak man, yet he believed him to be a great physician, and already, when Daniel was only five years old, he had promised him as a student. It was in consequence of this early pledge, that the son often went by the soubriquet of "Dr. Drake," long before he dreamed of what medicine was. Subsequently, however, it was determined that Daniel should remain at home, and study medicine with his cousin, Dr. John Drake, a young man of extraordinary promise, superior genius, and great moral purity. Just, however, as the period was approaching for the consummation of his plans, young Drake died, to the great disappointment of Daniel, who always regarded the event as a real misfortune, inasmuch as it deprived him not only of a good preceptor, but of the advantage of studying at home, and thus saving the expense of a residence at Cincinnati. His father, however, courageously persevered in his cherished purpose, although he himself would have preferred to learn the trade of a saddler, for which, in fact, he had already selected a master at Lexington, whither some of his cornfield companions had preceded him.

In the autumn of 1800, and at the close of his fifteenth year, he was sent to Cincinnati, and entered the office of Dr. Goforth, of that city, as a private pupil. The arrangement was that he should live in his preceptor's family, and that he should remain with him four years, at the end of which he was to be transmuted into a doctor. It was also agreed between the parties that he should be sent to school two quarters, that he might learn the Latin language, which, up to that time, he had, as already stated, wholly neglected. For his services and board, the preceptor was to receive $400; a tolerably large sum, considering the limited resources of his father.

During his pupilage, young Drake performed, with alacrity and fidelity, all the various duties which, at that early period of the West, usually devolved on medical students. His business was not only to study his preceptor's books, but to compound his prescriptions, to attend to the shop or office, and, as he advanced in knowledge, to assist in practice. The first task assigned him was to read Quincy's Dispensatory and grind quicksilver into mercurial ointment; the latter of which, as he quaintly remarks,* he found, from previous practice on a Kentucky

* Dr. Drake's Discourses before the Cincinnati Medical Library Association, p. 56, 1852.

hand-mill, much the easier of the two. Subsequently, and by degrees, he studied Cheselden on the Bones and Innes on the Muscles, Boerhaave and Van Swieten's Commentaries, Chaptal's Chemistry, Cullen's Materia Medica, and Haller's Physiology. These works constituted, at that time, the text-books of medical students, and the custom with many was to commit to memory the greater portion of their contents.

The term of his pupilage having expired, young Drake underwent the ordeal of an examination, and this being found satisfactory, his preceptor honored him with an autograph diploma, setting forth his attainments in the various branches of the profession, and subscribing it as Surgeon-General of the First Division of Ohio Militia. Under the sanction of this diploma, which he always greatly valued as a memorial of the olden time, and which was the first document of the kind ever granted in the interior valley of North America, Dr. Drake practised medicine for the next eleven years, when it was corroborated by another from the University of Pennsylvania.

In the spring of 1804, he formed a partnership with his preceptor, and in the autumn of 1805 he went to Philadelphia to attend his first course of lectures under the celebrated teachers, Rush, Wistar, Barton, Physick, and Woodhouse. At the close of the session he returned to the West. A journey, at that period, from Cincinnati to Philadelphia, usually occupied many days, often from twenty-five to thirty, and was generally performed on horse-back. As the roads were exceedingly rough, and the country was without bridges, it was always attended with great fatigue, and, sometimes, even with danger. Now the journey may be performed in a few days, the traveler lounging the while upon velvet cushions, and sleeping nearly as well as in his own bed.

Of his first preceptor, Dr. Drake always retained a lively and grateful recollection. In his "Discourses,"

delivered, in 1852, before the Cincinnati Medical Library Association, he gives a short but graphic account of him, from which it appears that he was a native of New York, that he emigrated early in life to Kentucky, and that he afterwards settled at Cincinnati, where he died in the spring of 1817, in the fifty-first year of his age. He was a man of the most winning and fascinating manners, was very kind and courteous to the poor, possessed fine conversational powers, with an inexhaustible fund of anecdote, was a warm politician, and was the first to practise vaccination in the West. Such a preceptor was worthy of such a pupil, and such a pupil of such a preceptor.

After his return from Philadelphia young Drake lived a year in Mason county, Kentucky, practising medicine, when he went to Cincinnati. Here he opened an office and gradually acquired business, but to what extent I am unable to say. While thus engaged, he married, in December, 1807, Miss Harriet Sisson, a grand-daughter of Col. Jared Mansfield, Surveyor-General of the Northwestern Territory, and afterwards a distinguished Professor in the Military Academy at West Point. This lady possessed elegant manners, unusual personal beauty, and a vigorous understanding. The union was a most congenial and appreciative one; their attachment, founded upon mutual esteem and good deeds, ripened with their years, and by degrees assumed almost a romantic character. In her counsel and sympathy, Dr. Drake found support and consolation in his pecuniary embarrassments, and in many of the other trials of his varied and checkered life. The issue of this union was three children, a son and two daughters; all of whom survive to inherit their parent's good name, and to transmit it unimpaired to their children. Mrs. Drake died in September, 1825.

He attended his second course of lectures in the University of Pennsylvania, in 1815, and was graduated at the end of the session, with the compliment from a member of the faculty of being a young man of great professonal promise! It will be seen hereafter that he was then already an author, having published, the preceding autumn, his celebrated "Picture of Cincinnati."

A little over a year after he received his medical degree, Dr. Drake was appointed to the Professorship of Materia Medica in the Medical Department of Transylvania University at Lexington, and in the following autumn entered upon the discharge of the duties of his Chair. His colleagues were Dr. Benjamin W. Dudley, afterwards so distinguished as a teacher and a surgeon, Dr. William H. Richardson, Dr. James Blythe, and Dr. James Overton, now of Nashville, Tenn. The number of students in attendance was twenty, of whom one, at the end of the session, received the honors of the doctorate. Dr. Drake, having completed his course, returned to Cincinnati to resume his practice, and the school was soon after suspended.

In 1819, Dr. Drake founded, at Cincinnati, the Medical College of Ohio, and immediately afterwards organized a Faculty, he himself taking the Chair of Medicine. A course of lectures was delivered to a small class of students, but misunderstandings soon sprung up, and Dr. Drake was expelled from the school by two of his colleagues, he himself being the presiding officer on the occasion.

Foiled in his attempt to build up a Medical Institution at home, he was induced, in the autumn of 1823, to re-enter Transylvania University, as an incumbent of the Chair which he had vacated six years before. He discharged the duties of this department with rare ability for two years; when, upon the resignation of Dr. Brown, he was transferred to the Professorship of Medicine, which he occupied until 1827, when he finally retired to Cincinnati; the number of pupils, in the meantime, having declined from 282 to 190.*

* Dr. Yandell's Introductory Lecture for 1852.

While quietly pursuing his practice, and editing the journal which he had established only a few years before, Dr. Drake was called, in 1830, to the Professorship of Medicine in the Jefferson College of Philadelphia; an institution founded only five years previously, and now, after the lapse of a quarter of a century, the first school, in the number of its pupils, on the Continent. Among his colleagues were two gentlemen whose reputation, then in an ingravescent state, became finally, like his own, coextensive with the American Union. I allude to the late Dr. George McClellan and the late Dr. Eberle, the one an ingenious and adroit surgeon, the other an able and accomplished physician. Both were excellent teachers of their respective departments, and both, but especially the latter, erudite and successful authors. It is no disparagement to these gentlemen to declare that the backwoodsman not only acquitted himself with great credit, but that, long before the close of the session, he was the most popular professor in the institution. His prelections, I well recollect, created quite a furor among the physicians of the city, as well as among the pupils in the University of Pennsylvania, not a few of whom wandered off, as the hour of their delivery approached, to her young, and then obscure, rival. Eloquence such as his, ready and off-hand, had not fallen from the lips of any teacher since the days of Rush; his manner, too, had something about it most winning and attractive; it was full of force, energy, and expression, and could not fail, of itself, to rivet the attention of the dullest intellect, while it was sure to captivate and charm the refined and cultivated. His success was complete; backwoodsism had triumphed, and henceforth medical men talked of Western teachers with more respect.

Had Dr. Drake remained in the Jefferson School no one can doubt that the brilliant success which has since awaited it would have been attained years before. With such colleagues as Eberle and McClellan, it would have rapidly risen into notice, and soon taken rank with the proudest institutions of the kind in the country. But such was not his wish ; he had other objects in view, and no sooner had the session terminated than he returned to Cincinnati. Previously, however, to doing this he organized a Medical Faculty in connection with the Miami University at Oxford, Ohio. But the scheme, which embraced two of his late Philadelphia colleagues, was not successful, and was finally abandoned before the commencement of the proposed lecture-term the ensuing autumn.

The Medical Department of the Miami University was evidently intended as a rival of the Medical College of Ohio, the fortunes of which had long been on the wane. The friends of the latter, perceiving the design, exerted themselves to effect an amalgamation of the two Faculties, and so far succeeded as to draw off a sufficient number of Dr. Drake's adherents to accomplish their object. To Dr. Drake himself was assigned a subordinate department, which, at the end of the session, he vacated, and once more retired to private life.

In the summer of 1835, Dr. Drake conceived the project of organizing the medical department of the Cincinnati College. He had, a short time before, been invited to the chair of medicine in the Medical College of Ohio, which he had founded sixteen years previously ; but believing that it would be impracticable, in the then existing state of things, to place the institution in a flourishing condition, he deemed it his duty to decline the offer, and to enter at once upon the business of establishing a new school. The first course of lectures was delivered the ensuing winter to a class of sixty-six pupils. The faculty consisted of seven members, with Dr. Drake at the head as professor of medicine. His colleagues were Dr. L. C. Rives, the present able and popular professor of obstetrics in the Medical College of Ohio ; Dr. Joseph Nash

M'Dowell, now of the University of Missouri; Dr. John P. Harrison, formerly of this city, and, after the downfall of the Cincinnati College, a professor in the Medical College of Ohio; Dr. James B. Rogers, afterwards professor of Chemistry in the University of Pennsylvania; and Dr. Horatio G. Jameson, a distinguished Surgeon of Baltimore, and at one time a professor in the Washington College of that city. To myself was assigned the Chair of Pathological Anatomy, at that period almost the only one of the kind in the United States. Most of these gentlemen had either belonged to medical Schools, or had been private teachers, and had thus already established a reputation as successful and popular instructors. Several, on the contrary, appeared before their pupils for the first time; but, notwithstanding this, they discharged their respective duties not only with credit to themselves, but to the admiration of their classes. At the close of the session Dr. Jameson resigned, and was succeeded by Dr. Willard Parker, the present justly distinguished Professor of Surgery in the College of Physicians and Surgeons of the city of New York.

With such a faculty the school could hardly fail to prosper. It had, however, to contend with one serious disadvantage, namely, the want of an endowment. It was, strictly speaking, a private enterprise; and although the citizens of Cincinnati contributed, perhaps not illiberally, to its support, yet the chief burden fell upon the four original projectors, Drake, Rives, McDowell, and myself. They found the edifice of the Cincinnati College, erected many years before, in a state of decay, without apparatus, lecture-rooms, or museum; they had to go east of the mountains for two of their professors, with onerous guaranties; and they had to encounter no ordinary degree of prejudice and actual opposition from the friends of the Medical College of Ohio. It is not surprising, therefore, that after struggling on, although with annually increasing classes, and with a spirit of activity and perseverance that hardly knew any bounds, it should at length have exhausted the patience and even the forbearance of its founders. What, however, contributed more, perhaps, than any thing else, to its immediate downfall was the resignation of Dr. Parker, who, in the summer of 1839, accepted the corresponding chair in the College of Physicians and Surgeons of the city of New York, an institution which he has been so instrumental in elevating, and which he still continues to adorn by his talents and his axtraordinary popularity as a teacher and a practitioner. The vacation of the surgical chair was soon followed by my own retirement and by that of my other colleagues, Dr. Drake being the last to withdraw.

During the four years the school was in existence it educated nearly four hundred pupils; the last class being nearly double that in the rival institution, an evidence at once of its popularity, and of the ability and enterprise of its faculty. The school had cost each of the original projectors about four thousand dollars, nearly the amount of the emoluments of their respective chairs during its brief but brilliant career.

Dr. Drake had the success of this enterprise much at heart, and often expressed regret at its failure; what the result might have been, if it had been vigorously prosecuted up to the present time, must, of course, remain a matter of conjecture. I have often thought, and so had my lamented friend, that we had vitality and energy enough in our faculty to build up a great and flourishing institution, creditable alike to the West and to the United States. He had a high opinion of the ability, zeal and learning of his colleagues, whom he never ceased to regard as one of the most powerful bodies of men with whom he was ever associated in medical teaching. The correctness of his judgment was amply confirmed by the elevated position to which most of them have since attained. Gradually one after another was called into the field, until all were at length employed in their former useful and honorable pursuits. Of that band, to which my heart and mind frequently revert, only four remain; three, after having attained an elevated position and reputation, slumber among the dead; while another is gradually and silently tottering into the grave which yawns beneath his feet.

Dr. Drake did not long continue idle. The Faculty of the Cincinnati College had hardly been disbanded, when he received an invitation from the Trustees of this University to the chair of Clinical Medicine and Pathological Anatomy. This chair, created with special reference to him, was not only novel in its character in this country, but it labored under the additional disadvantage of being an "eighth chair"; a circumstance without a precedent in the United States. The anomaly was still further increased by the establishment of an aggregate ticket of one hundred and twenty dollars. It was a bold experiment; but the result showed that those who made it had not acted in the matter unwisely. The new incumbent acquitted himself with great ability, the new chair soon became popular, and the rapid increase of the school fully attested the wisdom and the policy of the new measure, which secured to its faculty a gentleman of such enlarged experience and reputation as a teacher.

Dr. Drake remained in the occupancy of this chair until the spring of 1844, when, on the retirement of Dr. Cooke, he was transferred to the chair of Medicine. He continued to labor in this department with his accustomed zeal and eloquence until the close of the session of 1849; when he sent his resignation to the Board of Trustees. The winter before he vacated his chair he lectured to four hundred and six pupils, the largest class ever assembled within the walls of any medical institution in the valley of the Mississippi. The prosperity of the University, indeed, could hardly have been greater when he left it, although the number of students was somewhat less than the preceding session, and the utmost harmony prevailed in the Faculty. Notwithstanding these circumstances, he deemed it his duty to retire. The reason which he assigned for this step was, that he should, in another year, reach the period of life, when, by an act of the Board of Trustees, a professor becomes superannuated, and he thought it his duty to anticipate this law, notwithstanding the framers of it had, when they learned his intentions, abrogated it in his favor.

Soon after his retirement from Louisville, Dr. Drake was invited to the Chair of Medicine in the Medical College of Ohio; an appointment which, after some hestitation, he accepted, but which he filled only one session. Troubles, either real or imaginary, arose during the winter, and at the close of the term he found himself once more without a professor's chair. The introductory lecture which he delivered at the opening of the course is so characteristic of his love for the institution of his founding, and so expressive of his ardent temperament, that I cannot refrain from quoting from it one passage.

After alluding to his connection with various medical institutions, and to the fidelity with which he had served them; to the fact that he had been the first medical pupil in Cincinnati; and to the circumstance that he had founded, thirty years ago, the school in which they were then assembled, he says: "My heart still fondly turned to my first love, your alma mater. Her image, glowing in the warm and radiant tints of earlier life, was ever in my view. Transylvania had been reorganized in 1819, and included in its Faculty Professor Dudley, whose surgical fame had already spread throughout the West, and that paragon of labor and perseverance, Professor Caldwell, now a veteran octogenarian. In the year after my separation from this school, I was recalled to that; but neither the eloquence of colleagues, nor the greeting of the largest classes which the University ever enjoyed, could drive that beautiful image from my mind. After four sessions I resigned; and was subsequently called to Jefferson Medical College, Philadelphia; but the image mingled with my shadow; and when we reached the summit of the mountain, it bade me stop, and gaze upon the silvery cloud which hung over the place where you are now assembled. Afterward, in the Medical Department of Cincinnati College, I lectured with men of power, to young men thirsting for knowledge, but the image still hovered around me. I was then invited to Louisville, became a member of one of the ablest Faculties ever embodied in the West, and saw the halls of the University rapidly filled. But when I looked on the faces of four hundred students, behold, the image was in their midst. While there I prosecuted an extensive course of personal inquiry into the causes and cure of the diseases of the interior of the continent; and in journeyings by day, and journeyings by night — on the water, and on the land — while struggling through the matted rushes where the Mississippi mingles with the Gulf — or camping with Indians and Canadian boatmen, under the pines and birches of Lake Superior, the image was still my faithful companion, and whispered sweet words of encouragement and hope. I bided my time; and after twice doubling the period through which Jacob waited for his Rachael, the united voice of the Trustees and Professors has recalled me to the chair which I held in the beginning."

In the autumn of 1850, Dr. Drake was recalled to Louisville, to the chair which he had vacated eighteen months before. He remained in the school for two sessions, and then finally left it, once more to re-enter the Medical College of Ohio, now re-organized with an abler Faculty, and under brighter auspices. It was here, just at the opening of the session, full of hope and expectation about the class and the prospects of the Institution, that the hand of death was laid upon him, and that his varied but brilliant career was arrested.

The friends of Dr. Drake cannot but regret that he should have deemed it necessary, at his advanced age, to leave the University of Louisville, with which his name and fame had been so long associated, for the Medical College of Ohio. He could hardly have hoped, under the circumstances, to teach much longer, and it was scarcely reasonable in him to expect, that, in his endeavors to build up a great and flourishing institution, he could, at least for the first few years, enjoy much ease of mind, or relaxation of body. But a destiny seemed to have hung over him, and to have hurried him on. He could not, and would not, resist a long-cherished wish to spend the evening of his life in an institution, to which, early in his career, when he had not yet acquired any substantial fame, he had given birth. His affections had never been alienated from her for a moment, even in his exile as a teacher in other States; he fondly hoped that he should live long enough to see her assume a proud rank among the great schools of the country; and he prayed that God might permit him to breathe out his last breath in her service, and that he might die in the midst of her pupils, and be followed by them to his final resting place in the tomb. His wish, in this respect, was gratified; and few can doubt, that, had his life been spared a few years longer, he would have realized his other expectations.

Having spoken of Dr. Drake as a founder of Medical Schools, and of his connection with various Medical Faculties, we may, in the next place, contemplate him as a philanthropist and a patriot.

The subject of public education and morals was always near his heart. He took an active part in the establishment and support of the "Western Literary Institute and College of Professional Teachers" at Cincinnati, attended many of its meetings, often served upon its committees, and delivered several addresses, replete with wisdom and sound learning. The first time I ever heard him speak in public was at a meeting of this kind in 1834. He cherished, with a deep and abiding interest, all institutions for the diffusion of knowledge, and for the promotion of virtue and piety, as well as all charitable establishments, especially hospitals, lunatic asylums, and schools for the education of the blind and the deaf and dumb.

In 1821, he procured, by personal application to the Legislature of Ohio, aided by the recommendation of Gov. Brown, the establishment, at Cincinnati, of a charitable institution, denominated the Commercial Hospital of Ohio, of which, at the time of his death, he was one of the physicians. The grant was accompanied by an endowment, which has afforded the institution great facilities, and enabled it to diffuse its blessings widely among the poor-sick of the city and township of Cincinnati, as well as among the boatmen of the Southwestern waters. Connected with the Hospital was a Poor House, and an Asylum for the Insane; the latter of which, however, proving inadequate to the objects intended, Dr. Drake used every possible exertion, by repeated appeals to his brethren, and, finally, to the Legislature, to have this portion of the establishment removed, and placed under a separate board. The result was the present noble Institution for the Insane at Columbus, the Capital of Ohio.

In January, 1834, we find him addressing an appeal to the Legislature of his adopted State in behalf of the establishment of an institution for the education of the Blind; and, early in the following year, he read an able report before the Medical Convention of Ohio, at their meeting at Columbus, on the necessity for hospitals in the valleys of the Mississippi and the Lakes, for the accommodation and relief of those engaged in the commerce of the Southwest, as well as of travelers. Copies of this report were transmitted to the General Assembly of Ohio, and to the President of the United States, to Congress, and to the Heads of Departments. How far these labors were instrumental in promoting the object in question, I am not informed; but it is certain that Congress soon afterwards authorized the establishment of these institutions, and that they now greet the eye and cheer the spirits of the boatman at numerous points of the Southwest. It is but justice to state, in this connection, that the idea of this great and noble project originated with Dr. Cornelius Campbell, a benevolent physician of St. Louis.

In 1827, Dr. Drake established the Cincinnati Eye Infirmary. It was modeled after similar institutions in New York and Philadelphia, had a regular board of visitors, and was intended for the reception and accommodation of all classes of ophthalmic patients, the poor as well as the rich, but particularly the former. It was the first attempt of the kind in the Southwest, and, for a time, was eminently successful. The indigent sick from the city and neighborhood flocked to it daily for advice and treatment, and it speedily attracted persons from a distance. The consequence was that Dr. Drake soon became a distinguished oculist, and acquired no little skill as an ophthalmic surgeon. I doubt whether any other practitioner in the Southwest performed, during the first few years after the establishment of this institution, so many operations for cataract, artificial pupil, pterygium, and lachrymal fistula. His favorite operation for cataract was division, but he also occasionally performed extraction; a procedure requiring great manual dexterity and a thorough knowledge of the anatomy of the eye.

How long the Eye Infirmary remained open, I am unable to say; its usefulness was much impaired by the frequent absence of its founder, and after having been in a languishing condition for several years, its doors were finally closed. No attempt to revive the institution has since been made; a circumstance so much the more remarkable, considering the astonishing number of persons affected with all kinds of diseases of the eye in the Western and Southern States.

During his connection with this institution, Dr. Drake made ophthalmic medicine and surgery his special study; he purchased numerous works upon the subject, both in the English and French languages, and supplied himself with all the most approved instruments. Some of these works he reviewed elaborately in his medical journal; and he also published, about this period, several interesting and valuable papers upon different topics connected with this department.

To the influence of Dr. Drake was due, in no small degree, the establishment of the Kentucky School for the Instruction of the Blind in this city. The circumstances which led to the organization of this noble institution, so honorable to Louisville and to our State, are not without interest, and reflect much credit upon the zeal and benevolence of the subject of this memorial, who, admidst his vast professional labors, severe enough to daunt the hearts of fifty ordinary men, was ever keenly alive to the welfare and happiness of his fellow creatures of every rank and condition. In the winter of 1840—41, soon after he became a professor in this University, he delivered, once a week, upon the very spot upon which I now stand, a course of popular lectures on Physiology, and when he came to the eye, as an organ of vision, devoted an evening to the method of instructing the blind. With the aid of Mr. Patten, a former pupil of the School at Boston, he gave a practical illustration of the manner of teaching this unfortunate class of beings, in reading, writing, arithmetic and geography, concluding with an urgent and eloquent appeal to the audience, a highly numerous and respectable one, in favor of such an establishment in this city. The appeal, thus made, was not in vain. It was like a spark of fire falling upon combustible material. Every one present was affected by it; that evening, the blind, hitherto neglected, and almost forgotten, had many friends. Among these stood preëminent, as he always does in every charitable and benevolent enterprise in this community, one whose name is not less beloved than respected. I allude to our distinguished fellow-citizen, Judge Bullock. This gentleman, at the time referred to, was a member of the Legislature, and as soon as the lecturer sat down he came forward and expressed his determination to agita e the subject in the General Assembly, immediately after the opening of the session at Frankfort. Professor Drake afterwards furnished him with a variety of documents and reports, by the aid of which he framed a bill, which he sustained by an eloquent speech. Without difficulty it passed the house of which he was a member, but was laid over in the other.*

At the next session the bill was passed almost without a dissenting voice; the Legislature at the same time granting ten thousand dollars to endow the institution, on condition that this city would first raise the means to put it into operation. These means being promptly supplied, the school was organized forthwith. What progress it has made, what good it has achieved, and what blessings it is daily conferring, under the wise and benevolent management of its present superintendent, Mr. Patten, is too well known to require any comment on this occasion.

It would be doing injustice to my own feelings, as well as to the subject, if I were to neglect to mention, in connection with this topic, the names of Dr. Howe, of Boston, and Mr. Chapin, of Columbus, Ohio. These gentlemen, who are the superintendents, respectively, of the institutions for the education of the blind in Massachusetts and Ohio, benevolently visited Frankfort and Louisville, each with two pupils, with whose aid they gave several most interesting exhibitions, to the gratification both of the

* Western Journal of Med. and Surgery, vol. 5, p. 317 : 1842.

members of the Legislature and of the community at large. Such was the impression made by the exersises of these children upon the General Assembly, that, as has just been stated, Judge Bullock's bill was passed with hardly any opposition.

Dr. Drake had always, from an early period of his life, evinced a deep interest in the cause of temperance, unfortunately now so much on the decline. During his residence at Mays Lick, the rallying point, for many years, of the people of the neighborhood on election, parade, and gala days, as well as during court-time, he often had occasion, when yet a mere boy, to witness the deplorable and disgusting effects of the inordinate use of intoxicating drinks, and subsequently, after he had become a student and practitioner of medicine, he could not fail to observe that it was a frequent cause of disease and death, both moral and physical. He saw that it was the source of incalculable mischief, and that it lay at the foundation of nearly all the crimes that degrade and debase society, and reduce man to the level and condition of the animals by which he is surrounded. He saw at work an enemy, which, like "the pestilence that walketh by noon-day," silently but effectually destroys the peace and happiness of the domestic circle, which raises the arm of the parent against the child and of the child against the parent, and which fills our infirmaries, poor-houses, and penitentiaries with inmates. In a word, he saw that intemperance was sitting, like a mighty incubus, upon the bosom of society, tainting its very breath, and, in some instances, threatening the annihilation of entire families.

To such scenes, so well calculated to rouse his young and philanthropic mind, Dr. Drake could not long remain an idle and unconcerned spectator. He felt that there was a necessity for reform, and, like a true christian and patriot, as he was, he vigorously engaged in the work, determined, as far as his time and means would admit, to do his part in arresting an evil, fraught with such momentous consequences to the peace and happiness of his fellow-creatures. Address followed address, and for a time the pages of his medical journal, the sure and steady medium of communication between him and his professioal brethren, were literally teeming with articles upon the subject, dwelling with eloquent emphasis upon the malign and destructive effects of ardent spirits upon the human subject, considered in his moral, physiological, intellectual, and legal relations.

It was, while thus occupied in advocating and advancing the cause of temperance, that an incident occurred in the neighborhood of Cincinnati which afforded Dr. Drake an opportunity for the application of his knowledge and talents to the elucidation of a question of juridical medicine, often agitated but never until then fully established. In March, 1829, an old man, named Birdsell, was convicted on an accusation of the murder of his own wife, and sentenced to capital punishment. He had been long addicted to the inordinate use of ardent spirits, followed by occasional attacks of mania â potu, in one of which he committed the crime which he was about to expiate upon the gallows. Dr. Drake having carefully investigated the case, became so fully satisfied that the prisoner labored under a paroxysm of this kind at the time referred to, that he was induced to regard him as an irresponsible individual, precisely as a man who perpetrates homicide when affected with mental alienation from other causes. The court, however, waved all discusssion of the point, so ably presented by the learned witness, and submitted the case, with the broad facts, to the jury, who returned a verdict of murder in the first degree. A minute account of the trial was soon after published in the third volume of the Western Journal of the Medical and Physical Sciences, in which Dr. Drake fully elaborated his views, and unhesitatingly affirmed that insanity of this kind ought in law to be an immunity from punishment. The paper attracted much attention, and its sentiments received the unqualified approbation of a number of the leading medical men of the country. The American Jurist and Law Magazine, published at Boston, gave an extended notice of it, and endorsed the correctness of the author's conclusions ; a circumstance, which, considering the able character of that periodical, was highly flattering to his judgment and scientific attainments. Professor Beck, of Albany, also presented a full outline of the case in his great and learned work on Medical Jurisprudence, expressing his conviction of the correctness of Dr. Drake's opinion, and awarding to that gentleman the praise of originality for his suggestions. The case likewise attracted the attention of Gov. Trimble, who considered it of sufficient importance to invite to it, in his annual message, the attention of the General Assembly of Ohio, and who, when he found that his appeal was in vain, had the humanity, as well as the sagacity and firmness, to commute the punishment of the criminal into perpetual imprisonment ; thereby preserving the judiciary from the odium of illegally depriving a citizen of his life.

In December, 1841, Dr. Drake organized in this University, then the Medical Institute of Louisville, "the Physiological Temperance Society," for the benefit of the members of the medical class, of whom it was exclusively composed. Its object was to investigate the subject of alcoholic drinks, in their effects upon the system, and, incidentally, the abuse of other stimulants and narcotics. The society soon became popular with the pupils; for, in less than a month after its establishment , it had upwards of one hundred members, embracing nearly two-fifths of the entire class. Its meetings were held semi-monthly throughout the session of the school ; and its exercises, n which the distinguished and philanthropic founder, who was also its President, always took an active part, consisted in the reading of reports and the delivery of addresses on the nature and composition of the different kinds of liquor, and of their effects upon the system, in its healthy and diseased condition. The association continued in active operation until the Spring of 1849, when, in consequence of Dr. Drake's retirement from the University, it was abandoned. That its labors accomplished much good, and were frequently blessed in the persons of its members is too obvious to require any comment. The influence which it exerted was not confined to the pupils of the University, nor the members of the society; it extended over a wider sphere, and exhibited itself in every community in which its members settled.

In 1835, he exerted himself, with the ability of a statesman and the zeal of a true patriot, to enlist the attention of the people of the Southwest in favor of the establishment of a great railroad chain between the Ohio river and the tide-waters of the Carolinas and Georgia. In the month of August of that year, he presented an elaborate report upon the subject at a public meeting of citizens of Cincinnati, pointing out the advantages, in a commercial, social, and political aspect, of such a road, and concluding with an eloquent appeal to the people of the different States through which it was to pass, or which were to be benefitted by its erection. Great interest was, for a while, felt in the subject. On the 4th of July, 1836, a large convention was held at Knoxville, Tennessee, at which not less than nine States were represented. Dr. Drake was a member of that convention, as well as a member of the general committee which prepared business, and made a report on the practicability of the enterprise, and the best method of obtaining the requisite authority for carrying it into successful operation. The plan finally failed, chiefly on account of the unwillingness on the part of Kentucky, whose welfare, it was supposed, might seriously suffer by the result, to grant the right of way through her territory.

I was always of opinion, as indeed I am still, that Dr. Drake was exclusively entitled to the honor of having originated this gigantic scheme, so well calculated to develope the resources of the Southwest, and to furnish a means of intercommunication between her citizens, thus aiding in breaking down the bitter feelings of prejudice and hostility, growing out of the differences of their interests and domestic institutions; but I learned, some years ago, that another gentleman, since deceased, had set up his own claims, and defended them with some degree of acrimony. My information does not enable me to decide the question of priority in favor of either party. But I may be permitted to say that I was present at the meeting above referred to, at which my late friend read his report, and indulged in remarks tending to show that the credit of the suggestion belonged entirely to himself. Subsequently, in conversation with him upon the subject, he held similar language, and it is perfectly certain, as the proceedings declare, that he offered the resolution which led to the appointment of the committee of which he was the chairman. However the question may be ultimately decided by those most interested in it, it is much to be regretted that an enterprise, pregnant with such important results to the whole Southwest, and advocated by so able and zealous a champion, should never have been carried into effect for the want of proper co-operation of some of our States.

After having spoken of some of the advantages which would be likely to accrue from the undertaking, Dr. Drake makes the following pertinent remarks, the truth of which every one will acknowledge: "But the most interesting and affecting consequences that would flow from the execution of this enterprise would be the social and political. What is now the amount of personal intercourse between the millions of American fellow-citizens of North Carolina, South Carolina, and Georgia, on the one hand, and Kentucky, Ohio, Indiana, and Illinois, on the other? Do they not live and die in ignorance of each other; and, perhaps, with wrong opinions and prejudices, which the intercourse of a few years would annihilate forever? Should this work be executed, the personal communication between the North and South would instantly become unprecedented in the United States. Louisville and Augusta would be brought into social intercourse; Cincinnati and Charleston would be neighbors; and parties of pleasure would start from the banks of the Savannah for those of the Ohio river. The people of the two great valleys would, in summer, meet in the intervening mountain region of North Carolina and Tennessee, one of the most delightful climates in the United States; exchange their opinions, compare their sentiments, and blend their feelings; the North and the South would, in fact, shake hands with each other, yield up their social and political hostility, pledge themselves to common national interests, and part as friends and brethren." Noble sentiments! What heart is there that does not vibrate in unison with them, and devoutly wish for their consummation! The enterprise, if it had been executed, would have formed a great and enduring monument to the genius and patriotism of Dr. Drake, and served as a chain of adamant between the West and the South, identifying their interests, and binding them together by indissoluble ties. The Buckeye and the Palmetto would have intertwined their branches and kissed each other.

In a brief memorial, it cannot be expected that we should touch upon every event in the life of Dr. Drake. It is the duty of biography to elaborate his history and to place before the world, in a bold and conspicuous light, the prominent traits of his career. We cannot, however, close this branch of the subject, without bringing before you the recollection of a feature in his character, as extraordinary as it is beautiful and impressive. I allude to his attachment to Cincinnati, the checkered scene, for fifty years, of his labors and his usefulness. Although he was often absent, such was his loyality and devotion, that no earthly consideration could induce him to change his residence or abandon his citizen-ship. If he occasionally left her for a season, it was only that he might enjoy her the more at his return, as a lover sometimes voluntarily absents himself from his mistress that he may enjoy her presence the more at his reunion with her. His love for Cincinnati was real and unaffected. He had been her first medical pupil, her first medical graduate, her first medical author, and the founder of her first medical school. He had watched her progress with the satisfaction that a parent watches the career of a favorite and promising child; he had seen her in her weakness, and he had beheld her in the might of her strength, after she had risen to opulence and respectability as a great commercial mart, as a nursery of painters and sculptors, and as a city of able, enterprising, and enlightened men. If she is not the seat of a great medical school, an object which he had unceasingly at heart for the third of a century, the fault was not his, but of the circumstances by which he was surrounded, and which neither his genius, his industry, nor his tactics could control. Whether absent or present, whether in prosperity or adversity, he never ceased to love her, and to feel and manifest the deepest interest in her welfare and prosperity. There was hardly a measure, projected during his life-time, intended to promote her advancement, that did not either originate with him, or meet his hearty co-operation and support. Her people owe him a lasting debt of gratitude, not only for the many services which he rendered her, but also for being, at the time of his death, her greatest and most illustrious citizen.

His attachment to the West was hardly less remarkable.

No inducements could seduce him away from the adopted home of his parents; he loved its broad and luxuriant fields, covered with herds and wild flowers, its noble and romantic forests, rendered vocal with the music of birds and insects, and its graceful and majestic streams, bearing upon their bosom thrice a thousand vessels, freighted with the produce of its rich and fertile soil. Every thing around him was in harmony with his nature; and a residence in New-England, or New-Jersey, his native State, would have been as irksome and distasteful to him, as a residence at the Capital of the Union would be to the wild man of the forest.

Yet was his love not selfish; it was not limited to Cincinnati and the West; it embraced the whole Union, and gloried in every measure that was adopted for its safety, welfare, and perpetuity. A nobler and truer heart never pulsated in the bosom of an American citizen; a brighter and more steady flame of patriotism never burned in the breast of an American statesman. He watched with intense anxiety, hardly exceeded by that of Mr. Clay himself, the Compromise of 1850, and no one was more heartily rejoiced at its successful issue. While his domestic feelings, all his home sympathies, were for the West, his heart and soul were for the Union, embracing all its most cherished interests.

While the discussion of the Compromise question was going on in the Senate of the United States, and every where agitating the public mind, Dr. Drake was not idle. He had long perceived and lamented the ignorance which prevailed upon the subject of slavery in the Northern and Eastern states, and he determined, though not without reluctance, on account of the novelty of his position, to correct, if possible, some of the many misapprehensions under which many even of the better and more enlightend people of those regions labored. He knew, at all events, that an appeal to facts, vouched by his own experience and veracity, could do no harm, while, perhaps, it might effect some good. He could not disguise from himself the circumstance that his name was familiar to all the great and leading men of New-England, and he accordingly addressed himself, as the honored vehicle of his communications, to one of the fathers of his own profession in that country. This gentleman was Dr. John C. Warren, an old personal friend, by several years his senior, and for a long time professor of Anatomy and Surgery in Harvard University at Boston. The letters which he addressed to this distinguished physician and surgeon, were three in number, and they were published, some months afterwards, in the National Intelligencer at Washington. They were written in the winter of 1850–51, while the author was delivering a course of medical lectures in this University, and are characterized by great force of style, by remarkable moderation of tone and feeling, and by extraordinary logical precision, combined with a thorough knowledge of the subject. They attracted much attention at the time, and deserve to be preserved in book form, for extensive distribution. A copy should be sent to every house in the free states; for no better antidote could be furnished against the poisonous influences of such exaggerated productions as "Uncle Tom's Cabin." It would induce the rash and misguided to pause, and to consider whether the course they are pursuing is not calculated to do vast and abiding mischief, not only to the slave but to the Union. Dr. Drake deserves the thanks and the gratitude of every American citizen for stepping aside from his ordinary pursuits, from no other motive than that of serving his country, to discuss, in so able and philosophical manner, a topic of such great and absorbing interest.

I have heard it alleged that Dr. Drake was an abolitionist; an abolitionist in the modern and northern sense of the term. Such was not the fact. He was a man of too much intelligence, and too intimately acquainted with the subject of slavery, in all its forms and phases, to favor, in the most remote degree, the ill-judged, wild, and unfortunate movements of the northern fanatic. He knew that their tendency but too surely was to rivet the chains more firmly upon the colored man, and he had seen enough of slavery in his travels in the south to satisfy himself, that the condition of Africa's descendants was far better and happier than that of the free negro of the north, or even of the poorer classes of whites. Until the close of his fifteenth year, when he took up his residence at Cincinnati, he was an eye-witness to slavery in many of its worst forms. The early settlers of Mays Lick were severe task-masters; and numerous occasions occured in which his feelings were deeply shocked and his sympathies warmly excited. It was during his sojourn here, when he was hardly thirteen years of age, that the second constitution of Kentucky was adopted. During the canvass immediately preceding this event, a very strong effort was made over the whole State to elect members to the convention, who would favor the *gradual* abolition of slavery. Mr. Clay, who had only a short time before emigrated from Virginia, and settled at Lexington, united himself with that party, and labored zealously in the good cause; which, however, as is well known, failed of success. Dr. Drake's father was a warm and impassioned partizan; and his own feelings and thoughts ran in the same channel.

Subsequently, while he was connected with this University, and engaged in collecting material for his great work on the diseases of the Southwest, he had excellent opportunities for investigating the whole subject of slavery, and the result was, that he became satisfied that the condition of the negro in all the States which he had visited — Virginia, Kentucky, Tennessee, Missouri, Mississippi, Louisiana, Alabama and Florida — was far better than in former times; that he was better fed, clothed and lodged; less severely punished; and, in all respects, better cared for than he was forty, thirty, or even twenty years ago. In short, he saw every where around him evidences of amelioration and improvement, and a state of things strictly in harmony with the spirit and humane tendencies of the age. After having discussed the subject in all its bearings and relations, he concludes with the expression of his opinion that the policy and true interest of all is to leave all slaves and all negroes, except those of the north, to the management of the south.

Dr. Drake was a voluminous writer. His contributions to medical journals, in the form of original essays, reviews, and bibliographical notices, his temperance lectures, and public addresses, would, if collected, form several large octavo volumes. Much, indeed by far the most, of what he wrote was excellent; some was, perhaps, indifferent; but none was bad. "Nihil quod tetigit non ornavit." His style was always clear, fresh, and vigorous, often eloquent, and sometimes elegant. As a reviewer, his performances were generally rather analytical than critical. Indeed, as a critic he usually failed from a sense of too much cautiousness. As a medical journalist, he labored hard, and long, and zealously to elevate the character and dignity of the profession in the West and South, and did, beyond doubt, the cause an immense amount of service. His pen, for many years, was never idle; and if it was occasionally dipped in the ink of bitternes, to minister a rebuke or silence an enemy, it was only for a moment, when it would resume its wonted channel, and deposit the rich and varied freight of his well-stored mind.

His first attempt at medical or scientific authorship was in 1810, five years after he attended his first course of lectures in Philadelphia, and five years before he became a graduate. It was comprised in a small pamphlet on the "Topography, Climate, and Diseases of Cincinnati," where he then resided. Although designed exclusively for his professional and scientific friends, the work soon attracted the attention of travelers, in quest of information concerning the West, and thus suggested to him the idea of a treatise, constructed on a similar but much more extended scale. The result was his "Picture of Cincinnati," which soon acquired for him not only an American but a European reputation. It was published at Cincinnati in 1815, under the title of "Natural and Statistical View, or Picture of Cincinnati and the Miami Country." It was illustrated by maps, and accompanied by an appendix, giving an account of some late earthquakes, the aurora borealis, and Southwest wind; the whole forming a duodecimo volume of two hundred and fifty-one pages. The book soon attracted the attention of the public, and invited emigration to the West, but especially to Cincinnati, from all parts of America and Europe. Everybody became interested in a country, before so little known, and possessing advantages so glowingly depicted in the work under consideration. It was evident that the author had made a hit, not in a pecuniary point of view, but decidedly as it respected his reputation, and the future growth of what, in due time, was destined to become the "Queen City."

To give an analysis of this work would be out of place on this occasion. Suffice it to say that it is occupied with a geographical and historical account of the West, with a consideration of its physical, civil, political, and medical topography, and with a description of its antiquities, together with a notice of its projected improvements and future prospects, the latter of which are delineated in eloquent and patriotic terms, worthy of their and the author's high destiny. As a mere literary performance, the "Picture of Cincinnati" reflects the force and vigor of an able pen; but its pages are deformed by a bad style, a strange punctuation, and a profusion of dashes and commas, indicative of the writer's inexperi-

4*

ence as a practical scholar. Of these defects he himself seems to have been fully sensible, as he apologizes for them in his preface.

In 1827, twelve years after the publication of the "Picture of Cincinnati," Dr. Drake projected the Western Journal of the Medical and Physical Sciences, the first number of which appeared in April of that year. The motto of the work, engraved upon a flower of the Cornus Florida upon the title-page, was exceedingly happy and appropriate: "E sylvis, æque atque ad sylvas nuncius." It was literally, at that period, a messenger not only from, but also to, the woods. During the first year he had associated with him, as co-editor, Dr. James C. Finley, but at the end of that time it was brought out under his own management, which was continued until 1836, when, in consequence of his numerous engagements, and his frequent absence from home, he procured the efficient aid of Dr. William Wood, of Cincinnati, one of his former pupils. The Journal was originally issued monthly; but afterwards quarterly, and it continued to appear in this manner up to the period of the dissolution of the Medical Department of the Cincinnati College in 1839, when it was transferred to this city, and merged in the Louisville Journal of Medicine and Sugery, begun under the auspices of Professors Miller and Yandell, and Dr. T. S. Bell, but suspended before the completion of the first volume.*

It is no easy matter, even under the most propitious circumstances, to maintain a public journal of medicine. The difficulties were much greater twenty-five years ago than at present; then the West had few writers, and an editor was often compelled, from the paucity of material, to rely mainly upon his own efforts for filling up the pages

---

* Drake's Discourse before the Cincinnati Medical Library Association.

of his periodical. Many of the contributions that were sent in for the Western Journal of the Medical and Physical Sciences, displayed the most miserable scholarship, and the consequence was that not a few of them had to be entirely re-written before they could be committed to the hands of the compositor. "Copying, transposing, abridging, inverting, retroverting, decomposing, and recomposing," were a part of the labor and drudgery to which Dr. Drake had to submit in the progress of his enterprise. Nothing daunted, however, he worked hard upon its pages, which he adorned with many of his own effusions, both in the form of original articles and of reviews, until, after having been engaged upon it for twelve years, he finally, on his removal to Louisville, disposed of it in the manner already mentioned.

Writing nine years after the commencement of the journal, he quaintly observes that he had already owed allegiance to not less than nine publishers. "Thus," says he, "if our editorial vitality had not been truly feline, we should now be defunct." In consequence of these frequent changes the work was rarely issued with any regularity, and hence much complaint on the part of subscribers was the result.

The work received little or no patronage in the Middle and Atlantic States. When it had reached the completion of the sixth volume it had not a dozen subscribers on the other side of the Alleghany mountains. In Philadelphia, the seat of the editor's alma mater, and the so-called emporium of medical science, medical education, and medical scholarship, the only subscriber, for sometime, was the high-minded and public-spirited Mathew Carey, Esq., of that city. Nor was this all; the enterprise, it would seem, seldom received any notice from the medical press of the Eastern States; yet the work lived on, and attained quite a respectable age for one so entirely backwoods in its origin, style, and garb.*

The periodical having been transferred to Kentucky, now assumed the name of the Western Journal of Medicine and Surgery, which it still retains. Dr. Drake having acquired an extensive reputation as an able and indefatigable editor, consented to place his name upon the title-page, and thereafter the work was issued under the joint supervision of himself and Dr. Yandell. The senior editor, however, did not contribute much to its pages, for nearly all his time was now occupied in teaching and in collecting materials for his great work, and, in 1848, he finally withdrew from the enterpise. It is but just to add that for about four years, during which the editorship was in the hands of Dr. Drake, the journal received most important services from Dr. Colescott, of this city, upon whom much of its drudgery devolved, and who rendered the work somewhat famous on account of some of his caustic reviews.

The interest which Dr. Drake always felt for his profession induced him, in 1829, to begin the publication, in the Western Journal of the Medical and Physical Sciences, of a series of "Essays on Medical Education and the Medical Profession in the United States." The papers appeared in successive numbers of the periodical in question, and were finally, in 1832, collected into a small octavo volume of upwards of one hundred closely printed pages. They are written with the author's wonted vigor of style, and display, throughout, great sound sense, a discriminating judgment, and a profound acquaintance with the topics of which they treat. The number of essays amounts to seven; the first of which relates to the selection and preparatory education of pupils; the second to private

* West. Jour. Med. and Phys. Sciences, vol. 3, p. 152; 2d Hexade: 1833.

pupilage; the third to medical colleges; the fourth to the studies, duties, and interests of young physicians; the fifth to the causes of error in the medical and physical sciences; the sixth to legislative enactments; and the last to professional quarrels.

In looking, lately, with some degree of care, over this work, I have become impressed with the conviction that it is a production of great merit, and one that ought to be in the hands of every medical pupil and junior practitioner in the country. It comprises an admirable outline of medical ethics, or of the duties of medical men towards each other, of the responsibilities and requirements of the profession, and of the proper method of observing and investigating disease, conveyed in language at once forcible, dignified, and impressive. No one can rise from its perusal without sensibly feeling how much he has been instructed, and how far short he falls of the standard laid down by its distinguished author. It may be stated, as a remarkable fact, that the work covers the whole ground of medical education, and that it comprises every topic respecting medical reform so zealously, but indiscreetly, urged upon the consideration of the Ameriican Medical Association, at every returning meeting of that body.

In 1832, Dr. Drake published "a Practical Treatise on the History, Prevention, and Treatment of Epidemic Cholera," which was then desolating Cincinnati and the Western States. The work, forming a duodecimo volume of nearly two hundred pages, was designed both for professional and general use, and comprised an excellent and graphic account of that formidable malady; but it does not seem to have been well received, nor did it, I think add anything to the author's reputation. From the fact that much of it had been composed, and published in the Western Journal of the Medical and Physical Sciences, before he had witnessed the disease, it failed to inspire public confidence, and fell, in some degree, still-born from the press.

Two years after the publication of this treatise, he announced, as in progress of preparation, a work on "Anatomy, Physiology, and Hygiene," as a text-book for schools and colleges. The object was to promote the popular study of this branch of science, and to pourtray the pernicious effects of mere mental culture, without proper physical training. Some months after the announcement appeared he published a specimen of the style and arrangement of the book, and this was the last of it; for his leisure never permitted him to complete it. Some years after this he announced his intention of publishing a "Treatise on General Pathology," as a text-book for his pupils; but this also, for a similar reason, was never issued. The fact is that all his thoughts and affections were engaged upon his great work, and he regarded every thing else as of subordinate importance.

In 1842, Dr. Drake published, in the sixth volume of the Western Journal of Medicine and Surgery, a paper on the "Northern Lakes as a Summer Resort for Invalids of the South," which at the time attracted much attention from the medical and public press. The article, which had been previously read as an introductory address to his course of lectures in the University, was designed to illustrate the advantages offered, in the hot season, by our northern lakes, as a residence, to the people of the south, and was founded, mainly, upon his own observations made the preceding summer in a professional tour of two months. It abounds in beautiful and graphic delineations of the wild and romantic scenery of these great inland seas, of the towns and villages which stud and embellish their banks, of the nature of the climate, the productions of the surrounding country, the battle scenes of the late war with Great Britain, and the character and mode of life of the inhabitants, themselves a subject of study for the painter, the poet, and the philosopher. There are few tracts, of the same size, in the English language on the subject of travel, which contain so vivid, gorgeous, and life-like an account of the countries to which they relate. Nothing seems to have escaped the observation of the author. At one time his mind is dazzled and almost bewildered by a vast, dark, and impenetrable forest; at another, by the silvery and unruffled surface of a broad and unfathomable lake, reflecting the variegated and fantastic tints of the sky, or bearing upon its bosom the mighty steamboat, and the canoe of the adventurous Indian, the Canadian trapper, or the holy and self-denying missionary; now by some lofty and majestic cliff, rearing its head into the clouds, and serving as a monument of the works of God; and anon by the bewitching beauties of the setting sun, as his rays sport upon the heavens above, or paint, in all the gorgeous colors of the rain-bow, his image upon the waters below.

The latest of the minor productions of Dr. Drake's pen was a small volume of "Discourses," delivered, by appointment, before the Cincinnati Medical Library Association, on the 9th and 10th of January, 1852. It is comprised in a duodecimo volume of ninety-three pages, and is divided into two parts, the first of which treats of the early medical times in Cincinnati, and the other of medical journals and libraries. Few medical men, indeed few men of any profession, will rise from the perusal of this unpretending little volume without feeling that they have been both interested and instructed. The first part, giving an account of the pioneer physicians of the "Queen of the West," and of the prominent men and scenery of that early period, possesses, in my opinion, all the charm and interest of a romance, in which the lamented author, while he exhumes his predecessors and cotemporaries, and places them, in life-like colors, before the eyes of his readers, forms a prominent and conspicuous feature. His feelings were evidently deeply imbued with the spirit of the subject, and he has treated it in a style and manner of which no other man, either at Cincinnati or elsewhere in the West, is capable. It is replete with the characteristics of a man of feeling and genius. His similes and illustrations are so striking and forcible, that, in perusing this part of the book, the reader imagines himself in the veritable presence of the men and things which he delineates, and which pass, as in a moving picture, before him, even to the little Chickasaw pony, and the horrible witches, which at that early day still infested the neighborhood, and tormented the poor inhabitants! I doubt whether there is within the same compass of the "Pioneers," that most delightful romance of James Fenimore Cooper, so great an amount of powerful and graphic delineation of character, with so much true, artistic coloring.

On the occasion on which this address was delivered he looked around in vain for some of those professional brethren, whom, at the commencement of his career, nearly fifty years before, he had been in the hahit of meeting in the sick-room and at the social board. They had long since been removed from the busy scenes of life, and many of those who immediately succeded them, had shared a similar fate. "Like the living forms of an old geological era, they have," says the speaker, "become extinct, yet, as occurs with some species in geology, an individual has run into the later epoch, to mingle with its new and more perfect inhabitants. Of the little band behind the veil, I am the sole survivor; a sort of contingent remainder, bequeathed to the present generation, for any purpose to which so small a legacy may be applicable. For this length of days, I should humble myself before the Father of Life; but I may further manifest my gratitude by rescuing from oblivion the names of those who were my predecessors, and my compeers of that by-gone age."

At the close of the discourse he remarks: "I have thus given you some account of our past,—of the first third part of the life-time of our city profession. It was then in its infancy, and the foot-prints of childhood soon grew dim. Yet, if possible, this should not be allowed to fade quite away. Under this sentiment, I have used a little the chisel of Old Mortality; and feel that I have but discharged a duty, which, at all times, the present owes to the past; and the members of a generous profession to each other. Having been *pars minima* of what I have described, the verity of history required that I should not exclude myself. * * * * Every epoch of life should be allowed to illustrate itself. You yourselves will successively follow on. * * * At whatever age you may be gathered to your fathers, many of your plans will be left unfinished. I pray that the time may be far off; and, still more, that when it comes, each of you may be able, in faith, to lay hold of the cheering declaration of the inspired apostle—"Blessed are the dead who die in the Lord: they rest from their labors, and their works do follow them."

The second discourse exhibits an account of the origin and influence of medical periodical literature; and the benefits of public medical libraries. Time does not permit me to analyse it. It abounds in interesting and striking passages, and presents a full history of the periodical literature of the medical profession, both in America and Europe. At the close of it he exhorts his associates to constancy and perseverance in the enterprise thus auspiciously begun, and winds up with this eloquent passage: "The causes of failure generally lie in our own weakneses, of which the greatest is the want of unfaltering constancy. Holding on to the end in any laudable enterprise, is, with few exceptions, to achieve a triumph. I hope, and feel, and believe, that we *shall* steadily hold on; and thus, when some young student, now sitting thoughtful and silent in our midst, shall, with age and tottering footsteps, follow the mortal remains of the last of us to the grave, he will say to the physicians of another generation, then assembled around: "Carry forward the noble work which they began, make it better than you found it, and then hand it on to posterity."

The "Discourses" are dedicated to that accomplished gentleman, finished scholar, and eloquent teacher, Dr. Dickson, Professor of Medicine in the College at Charleston, South Carolina.

But the most splendid exhibition of his genius is in his work on the Diseases of the Interior Valley of North America, an enduring monument of his industry, his research, and his ability. Upon this production, which, unfortunately, he did not live to complete, he spent many of the best and riper years of his life. As early as 1822, in an appeal to the physicians of the Southwest, he announced his intention of preparing it, and solicited the coöperation of his professional brethren. His object, as stated in his circular, was to furnish a series of essays upon the principal diseases of this region of America, derived from his own observation and from that of his friends, and forming, when completed, a national work. Various circumstances conspired to delay the appearance of the work. The author's time, in the winter season, was much occupied in teaching, and in matters growing out of his official relations. Medical schools were obliged to be erected, fostered, and protected. Besides, he was the editor of a medical journal, to the pages of which he was often the chief contributor; and he was also frequently compelled to deliver public addresses, which consumed much of his leisure. His facility, in this respect, was too well known in the community, to permit him to remain unoccupied. The objects concerning which he was called upon to address his fellow-citizens were often of a benevolent character, and he had too much good nature to resist them, however much they might encroach upon his more legitimate pursuits and the great aim of his life.

In 1837, fifteen years after the publication of his circular, he found, for the first time, sufficient leisure to enter vigorously upon the collection of materials for his long contemplated work. In the summer of this year, accompanied by his two daughters, he visited a portion of the South for that purpose, during a tour of about three months. In 1843, he made a second tour, embracing Louisiana, Florida, Mississippi, Alabama, and the Gulf of Mexico; and subsequently he explored the interior of Kentucky, Tennessee, the two Carolinas, Virginia, Western Pennsylvania, New York, Illinois, Indiana, Michigan, Iowa, Wisconsin, Missouri, the great Lakes, and Canada. Wherever he went his fame preceded him, and he was kindly received by his professional brethren, many of whom vied with each other to show him attention and hospitality. It was during his absence upon these missions, which he performed with the zeal of an apostle of science, that he wrote those numerous and interesting traveling editorials, as he styled them, for the Western Journal of Medicine and Surgery. These epistles, which form so conspicuous a feature of that periodical during the time referred to, were usually descriptive of the manners, habits, and diseases of the people among whom he wandered, of the climate, scenery, and productions of the country, and, in short, of whatever seemed, at the moment, to strike his fancy, or interest his mind.

The materials thus collected were gradually digested and arranged, and finally presented to the profession, in the summer of 1850, under the elaborate title of "A Systematic Treatise, Historical, Etiological, and Practical, on the Principal Diseases of the Interior Valley of North America, as they appear in the Caucasian, African, Indian, and Esquimaux Varieties of its Population." The work, the first volume of which only has yet appeared, is illustrated by numerous charts and maps, designed and engraved at great expense, and was printed and published at Cincinnati under the author's immediate supervision. A second volume, the composition of which was nearly completed at the time of his decease, will be issued during the ensuing summer, under the care of a competent editor, and will be entirely devoted to subjects on practical medicine. The two together will constitute a monument of the genius and industry of their author, as durable as the mountains and the valleys, whose medical history they are designed to pourtray and illustrate. The toil and labor expended upon their production afford a happy exemplification of what may be accomplished by the well-directed and persistent efforts of a single individual, unaided by wealth, and unsupported by the patronage of his own profession.

It was originally the intention of Dr. Drake to comprise his work in two volumes; but as he progressed with the elaboration of his materials he found that he should have a sufficiency for another; which, now, that he is dead, will, of course, never be completed. The treasure remains, but the key wherewith to unlock it is gone.

It is to be regretted that the first seven hundred pages of the first volume were not published by themselves, apart from the matter which relates to the subject of febrile diseases, and with which they have no particular affinity. The arrangement was an unfortunate one, and is calculated to impair the sale of this portion of the treatise, which is better adapted to the taste and appreciation of such men as Humboldt and Malte-Brun, than to those of the medical profession, who seldom, at least, in this country, patronize works not strictly practical.

Every where at home the work recieved the most favorable commendation. All concurred in pronouncing it a performance of stupendous labor, and not a few of the leading journals declared that it was the most original production that our country had yet furnished. One of the most beautiful and appreciative notices of it appeared, soon after its publication, in the Daily Louisville Journal, from the pen of my colleague, Professor Yandell. Dr. Stillé, Chairman of the committee on Medical Literature, alluded to it in terms of high commendation in his report to the American Medical Association, at their meeting at Cincinnati, in May, 1850. Abroad its appearance was hailed with similar feelings. The British and Foreign Medico-Chirurgical Review, one of the ablest and most discriminating periodicals of the day, published a long and highly flattering notice of it. The veteran philosopher, Humboldt, to whom my colleague, Professor Silliman, took a copy, expressed his great delight at the work, and regarded it as a real treasure.

It would be premature to predict what estimate will be placed upon his works by posterity. That they will always be regarded as among the most valuable contributions of the nineteenth century, so prolific in noble productions, will not be doubted by any one at all familiar with the character of their contents. Truth is eternal, and whatever of this sublime essence they contain must be transmitted from one generation to another so long as man remains a civilized being, and possesses the faculty of communicatiug knowledge. Based as they are upon the observation of nature, and the just interpretation of her laws, they must always retain an ever-green beauty and freshness. They may become musty upon the shelf, the leaves may become stained, and their cover may become tarnished by age, yet will the student always find in them a never-failing source of instruction and enjoyment. Their author, like his illustrious name-sake, has performed a voyage of circumnavigation, which future scientific navigators may improve and extend, but which they must always follow as their chart and guide. Posterity will not fail to award him the enviable title of "Pioneer-Father of Medicine" of the Great Interior Valley of North America.

The works of Hippocrates, composed more than two thousand years ago, are as fresh to-day, and as much admired for their fidelity and truthfulness, as they were when they left the hands of the master-spirit that evoked them into existence. They have been translated into every language of Europe, and no one can read them without being sensible that the stock of his knowledge has been amplified and improved. The works of the American Hippocrates will be equally indestructible. Like his great protótype, he gathered their materials from the deep recesses, as well as from the surface of nature, and subjecting them to the plastic powers of his genius, he moulded them into a beautiful and harmonious whole, which proudly challenges our admiration and our gratitude. He has inlaid every line, and every sentence, and every paragraph with the skill of an artist, and the finish of an accomplished writer; his style is always clear and forcible, his language strong and pointed, his reasoning philosophical, his deductions just and logical, his arrangement of topics easy and natural. His composition, if not a model of elegance, abounds in examples of strong Anglo-Saxon vigor, which must strike and impress every sensible reader.

In studying the treatises of these two extraordinary men, one is sensibly struck with the similarity of the manner of their production. Both traveled extensively in their respective countries in quest of material for their works, both were close observers of nature, both are clear and forcible delineators of disease. Many of the events of the life of Hippocrates are involved in obscurity and fable. From some statements, however, contained in his treatises, it appears that he spent much of his time in travel, residing now at one place, and then at another, practising his profession, and studying the topography, climate, and diseases of the regions which he visited. In this manner he accumulated that rich store-house of facts which have transmitted his name to the present times, and which have laid the foundation, broad and deep, of medical science. The countries which he specially visited were Thrace, Athens, Delos, and Thessaly; but he penetrated many other regions, and it is generally supposed that he spent some time in Macedonia, in attendance upon the court of Archelaus.

The plan pursued by Drake was, in many respects, similar. He carried his perambulations, however, much further than Hippocrates, embracing a greater range and diversity of the human race, of climate, and diseases. We have already enumerated the names of the states and territories which he traversed in search of material for his work,

Other points of resemblance are to be found between these great men. Both were bold practitioners. Hippocrates laid much stress upon diet in acute diseases, and bled freely in cases of extreme pain and inflammation; sometimes opening two veins at once, to produce speedy fainting. Drake was a firm believer in the use of the lancet in the treatment of all acute inflammatory affections; his teachings upon this subject are not forgotten by his pupils, and all his friends know what confidence he placed in this remedy. Both practised Surgery. The practice of Hippocrates, in conformity with the times in which he lived, consisted of a few of the more simple processes of the art, as phlebotomy, cupping, and the application of the actual cautery; Drake, on the other hand, performed some of the more nice and delicate operations. We have already seen that he possessed more than common skill as an oculist.

The works of Hippocrates were collected and published after his death by his daughter. Drake issued one volume, but left the remainder incomplete, to be edited and published as a posthumous treatise. The works of great men would seem to resemble great cities, being always unfinished.

But while Drake resembled the father of physic in these respects he was unlike him in others. Hippocrates was descended from the Æsculapian family, under whose auspices and influence the medical school of Cos attained its high eminence and popularity. Drake, on the other hand, was the son of a poor, illiterate husbandman, whose ancestry, as far as it could be traced, was never distinguished for any achievements in medicine, law, divinity, politics, or arms. By the mother's side, Hippocrates was descended from Hercules; the mother of Drake had no such genealogy; all she could boast was that she was a good house-wife, and a kind-hearted, Christian woman. Hippocrates was rich; Drake was poor; the former possessed great scholastic advantages; the latter none; the one was educated early in life; the other comparatively late; Hippocrates enjoyed the benefit of the fame of his ancestors; it stimulated him, and brought him business; Drake, on the contrary, was the architect of his own fortune, and was obliged to depend for his advancement upon his own exertions.

I am not aware that Dr. Drake ever engaged in any purely literary composition, or that he ever contributed any thing to the literary periodical press, beyond some addresses and reports. For such pursuits he had no time, whatever might have been his fitness and inclination. Nor had he much leisure for indulging his taste in miscellaneous reading. Every moment of his time was occupied in lecturing to his pupils, in writing upon scientific subjects, and in laboring for the advancement of his profession, or the cause of morality and benevolence.

To his other accomplishments he added that of a poet. Several of his pieces, composed during the hours of relaxation from his professional pursuits, possess much beauty and sweetness. They generally partook either of the humorous, or of the solemn and pathetic. His "Parting Song," sung to the tune of "Auld Lang Syne" at the anniversary supper of the Ohio State Medical Society, at their meeting at Columbus, in January, 1835, and published in the eighth volume of the Western Journal of the Medical and Physical Sciences, is an ode of much beauty and feeling, and elicited the warmest applause at the time. It exerted the happiest effect upon every member present.

Dr. Drake was a man not of one, but of many characteristics. His very look, manner, step and gesture were characteristic; they were the outward signs of the peculiar nature within; his conversation, his voice, and modes of expression were characteristic; all tending to stamp him, in the estimation and judgment of the beholder, as an extraordinary personage. But there was one feature which jutted out, prominently and conspicuously, above and beyond the rest, and which served, in an eminent degree, to distinguish him from all the men of my profession I have ever known. This was intensity, intensity of thought, of action, and of purpose. This feeling, to which he was indebted for all the success which marked his eventful career, exhibited itself in all the relations of life; in his extraordinary devotion to his family, his attachments to his friends, his unfaltering love for his profession, in his interest in the cause of temperance, in his lectures before his pupils in the University, in his writings, his debates, and his controversies. No apathy or lukewarmness ever entered his mind, or influenced his conduct, in any scheme which had for its object the welfare of his species, the promotion of science, or the improvement of the human intellect. His temperament was too ardent to permit him, had he otherwise felt so inclined, to be an idle and unconcerned spectator of the world around him. It was not, and positive, like the pole of an electric battery, intense, ever restive, always doing.

It was this attribute of his mind which would have made him great and distinguished in any walk he might have chosen. He had talent and intellect enough, had he wished it, to have shone in the Senate, adorned the bar, or made a great pulpit orator. I have often thought that he had mistaken his profession, and that he ought to have been a politician. He might have made a great Secretary of State; for he had the astuteness of a Webster, the subtilty of a Calhoun, and the indomitable energy of a Benton.

His mind was quick, grasping, far-seeing; he acquired knowledge with great facility, sometimes almost intuitively, and readily perceived the relations and bearings of things. Embued with the true spirit of the Baconian philosophy, he delighted in tracing effects to their causes, and in unravelling the mysteries of human science and human knowledge. He was a keen observer, not only of professional matters, with which his daily studies brought him into more immediate contact, but of society and the world at large. Added to all this, he had a retentive memory, extraordinary powers of analysis, profound ratiocination, and great originality, with industry and perseverance seldom combined in the same individual. He possessed, in short, all the attributes of a great and commanding intellect, capable of vast exploits, and the accomplishment of great designs. His executive powers were extraordinary.

No where did this intensity exhibit itself in a more striking manner, or in a greater degree, than in the lecture-room. It was here, surrounded by his pupils, that he displayed it with peculiar force and emphasis. As he spoke to them, from day to day, respecting the great truths of medical doctrine and medical science, he produced an effect upon his young disciples, such as few teachers are capable of creating. His words dropped hot and burning from his lips, as the lava falls from the burning crater; enkindling the fire of enthusiasm in his pupils, and carrying them away in total forgetfulness of everything, save the all-absorbing topic under discussion. They will never forget the ardor and animation which he infused into his discourses, however dry or uninviting the subject; how he enchained their attention, and how, by his skill and address, he lightened the tedium of the class-room. No teacher ever knew better how to enliven his auditors, at one time with glowing bursts of eloquence, at another with the sallies of wit, now with a startling pun, and anon with the recital of an apt and amusing anecdote; eliciting, on the one hand, their admiration for his varied intellectual riches, and, onthe other, their respect and veneration for his extraordinary abilities as an expounder of the great and fundamental principles of medical science. His gestures, never graceful, and sometimes eminently awkward; the peculiar incurvation of his body; nay, the very *drawl* in which he frequently gave expression to his ideas; all denoted the burning fire within, and served to impart force and vigor to every thing which he uttered from the rostrum. Of all the medical teachers whom I have ever heard, he was the most forcible and eloquent. His voice was remarkably clear and distinct, and so powerful that, when the windows of his lecture-room were open, it could be heard at a great distance. He sometimes read his discourse, but generally he ascended the rostrum without note or scrip.

His earnest manner often reminded me of that of an old and venerable methodist preacher, whose ministrations I was wont to attend in my early boyhood. In addressing the Throne of Grace, he seemed always to be wrestling with the Lord for a blessing upon his people, in a way so ardent and zealous as to inspire the idea that he was determined to obtain what he asked. The same kind of fervor was apparent in our friend. In his lectures he seemed always to be wrestling with his subject, viewing and exhibiting it in every possible aspect and relation, and never stopping until, like an ingenious and dexterous anatomist, he had divested it, by means of his mental scalpel, of all extraneous matter, and placed it, nude, and life-like, before the minds of his pupils.

With abilities so transcendent, manners so ardent and enthusiastic, and a mind so well stored with the riches of medical science, Dr. Drake ought to have been universally popular as a teacher; nevertheless, such was not the fact. First course students often complained that his lectures were abstruse, in a degree wholly beyond their comprehension; that they could not follow his reasoning, or argumentation, and that, despite their best directed efforts, they were unable to derive much profit. The more advanced members of his classes, on the contrary, never experienced any such trouble; they felt the deepest interest in every thing that he uttered, and never failed to look upon him as a most able and instructive teacher. To account for this discrepancy it is necessary to state that Dr. Drake's method of instruction differed materially from that of most of his cotemporaries, both in this country and in Europe. Instead of beginning his course with the practical, every-day details of his department, he always devoted the first six weeks to the inculcation of general principles, deeming a knowledge of them of paramount importance to every student of medicine. This he always regarded as the *philosophical* part of the course, and he spared no efforts to place it prominently before the minds of his pupils. In doing this he was fully conscious of the difficulty under which he labored, and often lamented, in bitter strains, the deficiencies, on the part of his classes, which prevented them from appreciating his instruction. He saw how little many of the youth who resort to our lecture-rooms are prepared, by ther habits and education, to profit by such a mode of teaching; and yet he could not, durst not, in conformity with the dictates of his conscience and judgment, pursue any other. He would rather be unpopular with a portion of his classes than sacrifice duty and principle, or deviate from the standard which he had adopted as the rule and guide of his conduct in the lecture-room.

His fluency and facility of language gave him great advantage as a public debater. To his ability as a profound reasoner, he added subtilty of argument, quickness at repartee, and an impassioned tone and style, which rarely failed to carry off the palm in any contest in which he was engaged. During his sojourn in Philadelphia in 1830–31, Broussaisism, then so fashionable in that city, formed the subject of discussion before the Philadelphia Medical Society. Being a member, he was induced to take part in the debate against the doctrine, while Dr. Jackson, the present Professor of the Institutes of Medicine in the University of Pennsylvania, and himself no mean speaker, arrayed himself on the opposite side. The discussion, which had been in progress for several evenings, waxed warmer and warmer, and excited universal interest among the members and visiters. Dr. Drake had the floor, and had already made a brilliant effort, when, suddenly stopping, he exclaimed, in a loud voice, and with peculiar emphasis, addressing himself to the chairman, the distinguished Dr. Condie, "Sir, is it not so?" The effect was electric. The worthy dignitary, unconscious apparently of what he was doing, sprang upon his feet, exclaiming, "Yes sir, I believe it is," to the great amusement of every one present.

Dr. Drake always manifested extraordinary interest in the moral training of medical pupils. Sensible of the temptations which constantly beset their path, and allure them from their duty, he took special pains at the opening of every session of the different schools with which he was, from time to time, connected, to point out to them their proper position, and to warn them of their danger. As a means of promoting this object, as well as of advancing the respectability of the profession, he delivered, while a professor in the Cincinnati College, for several winters, a series of Sunday morning dicourses to the students of that institution, on medical ethics, the *morale* of the profession, and the virtues and vices of medical men, embracing their duties to their patients, to the community, and towards each other. These addresses were usually attended by large numbers of the citizens of Cincinnati, and exerted a wide and happy influence upon the youths for whom they were more especially prepared. Their publication would, I doubt not, be well received by the profession.

In this University, as was before stated, he interested himself greatly in promoting the cause of temperance among the students; and, as a means of religious improvement, he was in the habit, for many winters, of joining such of them as felt an interest in the subject, at a Sunday morning prayer meeting. In a word, he was ever ready with his advice and kindly offices, often affording aid and comfort to those who, in the absence of their parents and guardians, stood in need of a counsellor and friend.

He had a decided taste for the society of the young men of his profession, and always evinced a deep interest for their prosperity. The instances were not few in which he labored to advance the welfare of young men, some of whom have since risen to deserved distinction. It was his lot, especially in the earlier periods of his life, to have numerous private pupils, several of whom he educated gratuitously, at the same time treating them with true parental regard and tenderness. As a public teacher in the different schools with which he was connected, he aided in educating several thousand young men, and fitting them for the practical and responsible duties of their profession. The seed thus sown has brought forth much good fruit, the happy effects of which will be felt in future generations.

His own standard of medical knowledge was of the most elevated nature. No one understood better than he the importance of a thorough education, and of a well disciplined mind. His own early deficiencies, ever present, and ever recurring, had made an impression upon him, which nothing could efface. His occupation as a teacher of medicine had brought him, for years, in daily contact with men and youths, who were not only destitute of preliminary education, but absolutely, from the want of opportunity, and mental capacity, utterly incapable of acquiring any. This state of things, so prevalent and deplorable, he often lamented to his friends and colleagues, while he never failed, on all proper occasions, to assail it in his writings and prelections. The difficulty under which a teacher labors in imparting instruction to such pupils, and preparing them for the successful exercise of their high and responsible duties, as practitioners, can be more easily imagined than described. His daily experience in the lecture-room showed Dr. Drake how much of the good seed that is there sown falls upon barren soil; or how, instead of producing good fruit, it yields nothing but tares and thorns. Such was his feeling upon this subject that in numerous conversations he had with me respecting it, he often expressed himself as being almost ready to abandon teaching forever. Like many others, he perceived the remedy, but was unable, from the want of coöperation, to apply it. Poor as he was, he would a thousand times rather have lectured to a hundred intelligent and well-prepared young men than to five hundred ignorant aud ill-prepared. His object was not the acquisition of gain, but the desire to be useful and profitable to those whom it was his duty to instruct in the great principles of the healing art.

Of quackery, in all its forms and phases, he was an uncompromising enemy. He loved his profession and the cause of truth too well to witness, without deep solicitude, its impudent and unhallowed assaults upon the purity and dignity of medicine, considered as a humane, noble, and scientific pursuit. Hence, he permitted no suitable opportunity to pass without rebuking it, and holding up its advocates to the scorn and contempt of the public. In common with many of his brethren he deprecated its unblushing effrontery, and regretted the countenance and support which it derives from a thoughtless clergy and an unscrupulous and unprincipled press. He saw that it was an evil of great magnitude, threatening the very existence of our profession; and, as a journalist, he deemed it his duty to bring the subject frequently and prominently before his readers, intreating their aid and coöperation in suppressing it. How well he served the cause of his profession, in this respect, those only who are acquainted with his labors are competent to judge.

He was the founder of no new sect in medicine. For such an enterprise he had no ambition, even if he had been satisfied, as he never was, of its necessity. He found the profession, when he entered it, at the dawn of the present century, steadily advancing in its lofty and dignified career, refreshed, and, in some degree, renovated, by his immediate predecessors, and his chief desire was to ingraft himself upon it as an honest, conscientious, and successful cultivator. How well he performed the part which, in the order of Providence, he was destined to play, in this respect, the medical world is fully apprised. No man was more sensible than he of the imperfections and uncertainties of the healing art, and no one, in this country, in the nineteenth century, has labored more ardently and zealously for its improvement. For the systems of the schools no physician and teacher ever entertained a more thorough and immitigable contempt. He was an Eclectic in the broadest and fullest sense of the term. His genius was of too lofty and pervasive an order to be trammelled by any authority, however great, respectable, or influential. Systems and system-mongers were alike despised by him, as they could not, in his judgment, be otherwise than dangerous in their practical bearings, and subversive of the best interests of science. It was Nature and her works that he delighted to study and to contemplate; not that he regarded with indifference whatever was good and valuable in the productions of others, but simply because he preferred to drink at the fountain instead of at the turbid stream. Like Hippocrates and Sydenham, he was a true observer of nature, and, we may add, a correct interpreter of her laws and phenomena; his ambition was to be her follower during life, and at his death to leave a record, a true and faithful transcript, of the results of his investigations for the benefit of his brethren.

In his intercourse with his professional friends his conduct was a model. His code of ethics was of the purest and loftiest character. He was not only courteous and dignified, but eminently considerate of the rights of others; his habits of punctuality were established early in life, and were never departed from. He made it a rule never to make a professional brother wait for him at a consultation. When the appointed moment arrived, he was at his post, ready to enter upon the business before him. His deportment, on such occasions, towards his juniors, was always conciliatory, and, at times, even defferential. It was, apparently, a study with him to make them feel at ease, that they might deliver their views and opinions freely, and without embarrassment. If he differed in sentiment, concerning a point of pathology, diagnosis, or practice, he expressed himself with the modesty of a gentleman, and the kindly feelings of a professional brother. He was as free from hauteur and assumption as he was from affectation and pedantry, which no man more despised.

The examination of his cases was conducted with great care and attention; indeed, he seemed occasionally to be over-minute and even tedious, spending a longer time over his patients than the exigencies appeared to require. His early habits of caution never forsook him at the bedside of the sick.

In his intercourse with his patients his conduct was regulated by the nicest sense of honor. No one understood better how to deport himself in their presence, or how to preserve inviolate their secrets. Hippocrates, who exacted an oath from his pupils never to reveal any thing that was confided to them by their employers, never more scrupulously observed the sanctity of the sick-chamber. Kind and gentle in his manners, he was as much the friend as the physician of his patients, not a few of whom made him their confidant and counsellor. The advice which he delivered under such circumstances was often of great service to the interested party, by whom it was never forgotten, owing to the earnest and solemn tone in which it was imparted.

In the bestowment of his time and labor, he made no distinction between the rich and the poor; the latch-string of his heart was accessible to all. "The importance of the malady, and not the patient's rank or purse, was the measure of the attention which he paid the case." It was his province, from his peculiar relations, to attend gratuitously, long after he had attained the most exalted rank in his profession, numerous widows and orphans, as well as the families of not a few of his old friends, who had become poor in consequence of the vicissitudes of fortune. These labors, which encroached much upon his time and domestic enjoyments, he always performed with a willing heart, ever regarding their objects as specially entitled to his consideration and regard.

I had great confidence in his professional acumen; I saw enough of him in the sick chamber to satisfy me that he had a most minute and thorough knowledge of disease, and of the application of remedial agents. There was no one whom I would rather have trusted in my own case, or in that of a member of my family; yet there were some, and some in our own profession, who pretended to have no confidence in his judgment or skill, who thought him a mere theorist, a bold, closet speculator, and an unsparing, reckless practitioner, whose treatment was altogether too spoliative, and, consequently, dangerous. Of the truth of such a charge, I never, during a familiar acquaintance with him of many years, had any evidence. The charge, doubtless, had its origin in jealousy and misconception. It can hardly be supposed that a man of such transcendent intellect, who had studied his profession so well, so anxiously, and so intensely, who had observed disease so long and so thoroughly, who had written so much, and delivered so many courses of lectures; in a word, who had devoted his whole life to the science of medicine and its kindred branches, should have been a bad or even an indifferent practitioner. The idea is too absurd to require serious refutation. It is abundantly disproved by the fact that those who knew him best had generally most confidence in him in this respect. Many of his most intimate friends in Cincinnati continued to employ him up to the latest period of his life, as did also not a few of his earlier patients; persons who may be presumed to have been fully competent to appreciate his judgment and practical ability. He rarely gave a lecture in this University without, at the close of it, prescribing for five or six of his pupils. An hour was thus not unfrequently spent every day of the week.

Besides, he never lacked business; in the early part of his career his practice was large and laborious, and if, as he advanced in age, it became comparatively small, it was owing, not to a want of confidence on the part of the public, but to his frequent and protracted absence from Cincinnati; a circumstance wholly at variance, in every community, with the acquisition and retention of a large family practice. Such a practice, in fact, especially as he grew older, he did not desire; it was incompatible both with his inclinations and the great object of his ambition, which was to teach medicine, and to compose a great and useful work on the diseases of the interior valley of North America. Business never forsook him at home or abroad; the numerous letters which he received from his professional brethren and from patients at a distance, soliciting his advice in cases of difficulty and doubt, show in what estimation his science and skill were held by the public.

His practice in acute inflammatory diseases was bold and vigorous. The lancet was his favorite remedy, and he drew blood freely and without stint in every case in which the symptoms were at all urgent or threatening, provided the system was in a condition to bear its loss. Having attended, in early life, the lectures of Dr. Rush, the most eloquent and captivating teacher of medicine in his day, in this country, and a strenuous advocate of sanguineous depletion, he imbibed a strong prejudice in favor of this practice, which he retained to the latest period of his career. But it would be unjust to say that he employed the remedy without judgment or discrimination. If he bled freely he also knew when to bleed. No man had a better knowledge of the pulse and the powers of the heart.

His conduct, in all the relations of life, was most exemplary. In his friendships, usually formed with much caution, he was devoted, firm, and reliable, as many who survive him can testify. His attachments were strong and enduring. Few men, as he himself declared to me only a few months before his death, possessed so many ardent and faithful friends. His social qualities were remarkable. He loved his friends, enjoyed their society, and took great pleasure in joining them at the domestic board; where, forgetting the author and the teacher, he laid aside his "sterner nature," and appeared in his true character, plain and simple as a child, cheerful, amiable and entertaining. It was during such moments, which served to relax the cords of his mind, and fit it for the renewal of its labors, that he shone to most advantage. His conversational powers on such occasions, as well as in the drawing-room, although superior, were not equal. Like all great and busy men, he had his cares and annoyances, his hours of depression and despondency, his fits of absence and restlessness.

We have stated that Dr. Drake had many warm, staunch, and admiring friends. It would be untrue to say that he had no enemies. He had too ardent and positive a temperament, too much ambition, too much intellect, to be altogether exempt from this misfortune, if such, indeed, it may be called. I assume, and I think the world's record abundantly confirms the conclusion, that no great, useful, or even truly good man was ever wholly without enemies. Such an occurrence would be an anomaly in the history of human nature. It has been well observed by one who was himself great, and who occupied, for many years, no small space of the public eye, that "slander is the tax which a great man pays for his greatness." The more conspicuous his position the more likely will he be to have enemies, to assail and misrepresent his character. It is only the passive, the weak, the idle, and the irresolute who are permitted to pursue, unobserved and unmolested "the even tenor of their way." To this class our friend did not belong. His mission was a higher and a nobler one. He was destined, under the arrangements of Providence, to perform great deeds, and to be a great and shining light in his profession, and it would have been just as impossible for him, in attempting to carry out these designs, to steer clear of enemies, as it was for the three great and illustrious statesmen, whose loss has been so recently bewailed by a nation's tears, to fulfil their great mission, and live and die in peace with the men and parties whose interests, and passions, and prejudices they were constantly obliged to assail and combat.

But to the credit of Dr. Drake let it be said that he never wilfully wronged any human being. He was always just, always truthful, always conscientious. He never struck a blow where none had been struck before. Men who are fond of using harsh expressions have accused him of being captious, over-bearing, dictatorial. During an acquaintance, intimate and uninterrupted, of nearly twenty years, during most of which we were colleagues at Cincinnati and in this city, I never witnessed any exhibition calculated to confirm such an accusation. His official relations in this University, as my associates here can testify, were of the most agreeable and harmonious nature. There was not a measure, intended to advance the prosperity of the institution, proposed by his colleagues, or the Honorable Board of Trustees, that did not meet with his hearty concurrence and support. There was no one whose pen was more frequently and effectively employed in repelling the assaults of its enemies, in encouraging the lukewarm, and in cheering the friendly.

His early associations in Medical schools, particularly in the Medical College of Ohio, his first and last love, were unfortunate, and exerted for a long time, if not, indeed, during the rest of his life, an unhappy influence upon his reputation as a quiet and peaceable man. Many of his colleagues were ordinary individuals, either wholly unfit for the discharge of the responsible duties assigned to them by the nature of their chairs, or, at all events, ill-calculated to aid in building up a great and flourishing school. Misconceptions, misrepresentations, and, finally, bitter and unrelenting quarrels were the consequence of this connection, which, from the attitude in which he was always placed as the prominent party, generally fell with severest effect upon Dr. Drake. Thus he was often made to occupy before the profession and the public a false position, and obliged to act a part which did not naturally belong to him. It seems to have been a principle with him, at this period of his life, never to allow a charge uttered by an assailant against his character to pass unnoticed or unrebuked. So frequent were these missives that, at length, even some of his warmest and most intimate friends were disposed to look upon him as a bitter and unrelenting controversialist. Nothing, however, could have been more unjust. His great error was that he was morbidly sensitive, and that he permitted himself to be annoyed by every puff of wind that swept across his path. Baseness and malignity never entered into his character. In all his difficulties and troubles, growing out of his early professional relations, I know not a solitary one in which he had not strict justice on his side. Nature and art had combined to give him powerful weapons, and no man better understood how to use them against the assaults of his enemies.

Of all his *early* associates in the Medical College of Ohio, Dr. John D. Godman was almost the only one for whom he cherished any sincere respect, or who came up to the standard he had formed of what a colleague and a teacher ought to be. That standard was, perhaps, capriciously high, so elevated as to render it difficult for any but a favored few to attain it. Be this as it may, it was, I doubt not,the cause of many of the troubles in which he was so soon to be involved, and which fate, blind and ill-directed, seemed ever ready to recall and perpetuete. For Godman, his first colleague in the chair of Surgery in the Medical College of Ohio, rocked, like himself, in the cradle of poverty, deprived, like himself, of early educational advantages, and set apart, like himself, for some mechanical pursuit, he ever cherished the warmest friendship and the most tender regard. He was evidently a man after his own heart, pure, of lofty ambition, full of genius and industry, and bent upon the achievement of great designs. How well he succeeded the history of his short, sorrowful, and not uneventful life bears abundant testimony. Compelled to work for nearly two years with only one lung for his daily bread, he performed, even while in this crippled and dying condition, an amount of labor which may well put to shame the puny and flickering attempts at support and reputation of the Young Physic of the present day, so vaunting, and yet so unproductive of useful and profitable results. Cut off at the age of thirty-seven, by a ruthless disease which never spares its victim, he had acquired a character for purity of conduct, varied learning, and profound attainment as an anatomist and naturalist, as rare as it was extraordinary and wonderful. It seems as if nature had offered him as an example of what a man, supported by talents, industry, and singleness of purpose, may accomplish in the shortest time, under circumstances the most adverse, and under trials which, to ordinary mortals, appear insurmountable. Godman expired at Germantown, Pennsylvania, on the 17th of April, 1830. His friend was not unmindful of him after his death. In the fourth volume of his journal, he paid a beautiful and touching tribute to the memory of one who was, in every respect, worthy of his esteem and affection.

(TO BE CONCLUDED.)

